# Supplementary material for: The impacts of active and self-supervised learning on efficient annotation of single-cell expression data
Source: Nat Commun. 2024 Feb 3;15:1014. doi: 10.1038/s41467-024-45198-y (PMC10837127; doi:10.1038/s41467-024-45198-y)
Supplement: Supplementary file 1 — Supplementary Information [file 41467_2024_45198_MOESM1_ESM.pdf]

Supplemental tables

	Parameter	Possible values	Parameters space
**Models**			
Scmap	Classification level	• Cell level<br>• Cluster level	2
SingleR	Default		1
RandomForest	Internal grid search over parameters		1
CyTOF-LDA	Default		1
SVM-rejection	As described in[56]		1
SingleCellNet	Default		1
**Subtotal**			**7**
**Cell selection**			
Active learning	Initial cell selection	• Random<br>• Ranking	2
	Entropy uncertainty	• Highest entropy<br>• 95% entropy<br>• 75% entropy	3
	Maxp uncertainty	• Lowest maxp<br>• 5% maxp<br>• 25% maxp	3
**Subtotal**			**12***
Adaptive reweighting	KNN parameter	• 10<br>• 20<br>• 30	3
	Clustering resolution	• 0.4<br>• 0.8<br>• 1.2	3
**Subtotal**			**9**
Random selection	Repeated 3 times		3
**Subtotal**			**3**
**Total**			**168****

Supplemental table 1. Shown is the total parameter space explored for a single train-test split and modality of our benchmarking pipeline.

* The total is 12, as for each entropy and maximum probability selection metric each cell selection is run, thus the total is 3*2 + 3*2 = 12.

** The total number of jobs per dataset split is the number of cell selection methods times the number of models fit: (12*7) + (9*7) + (3*7) = 168.

Supplemental figures

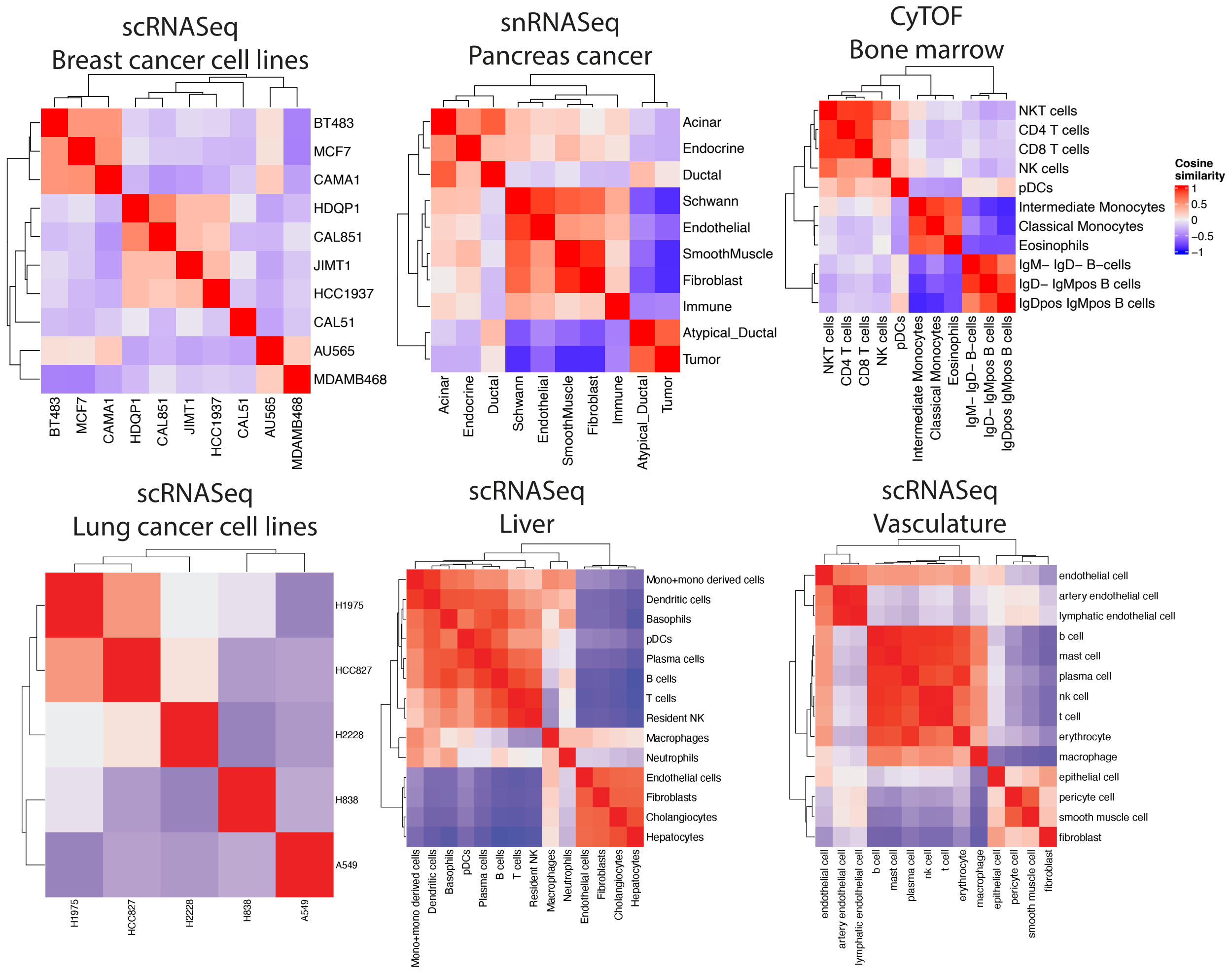

S. Figure 1. Cell type similarities for all cohorts. Weighted cosine similarity values between all cell types for all datasets. Source data are provided on zenodo: https://doi.org/10.5281/zenodo.10403475.

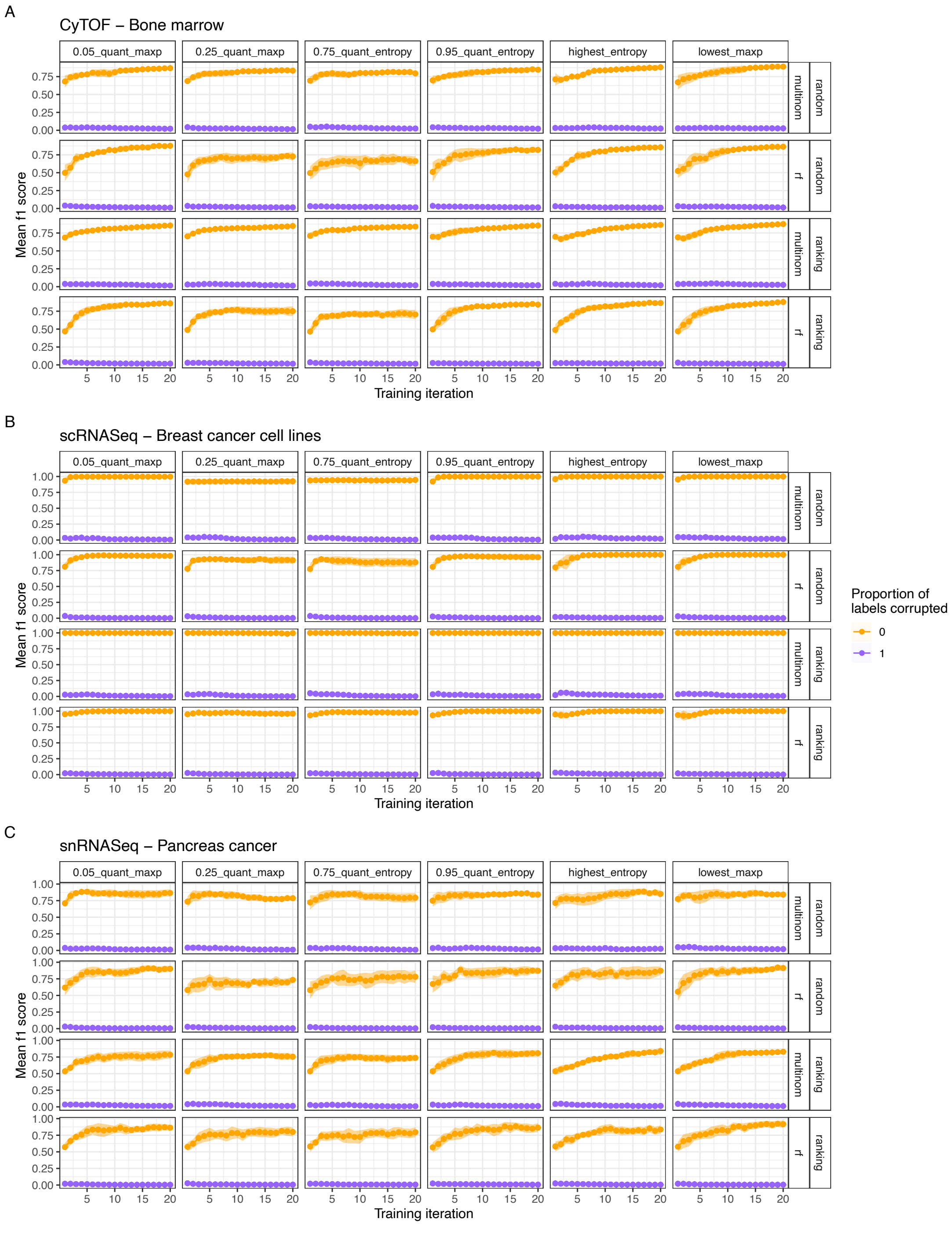

S. Figure 2. Active learning algorithms work as intended. A-C) Accuracy of the active learning classifier measured using the average F1-score across 10 seeds as a function of the active learning iteration. As a control, all training labels were corrupted (purple). The columns show the results for each active learning setting, while the rows show how the initial set of cells was selected (randomly or ranked) and what active learning model was used (random forest, rf or logistic regression, multinom). The same plot is shown for each cohort: A) CyTOF - Bone

marrow, B) scRNASeq - Breast cancer cell lines and C) snRNASeq - Pancreas cancer. Source data are provided on zenodo: https://doi.org/10.5281/zenodo.10403475.

A

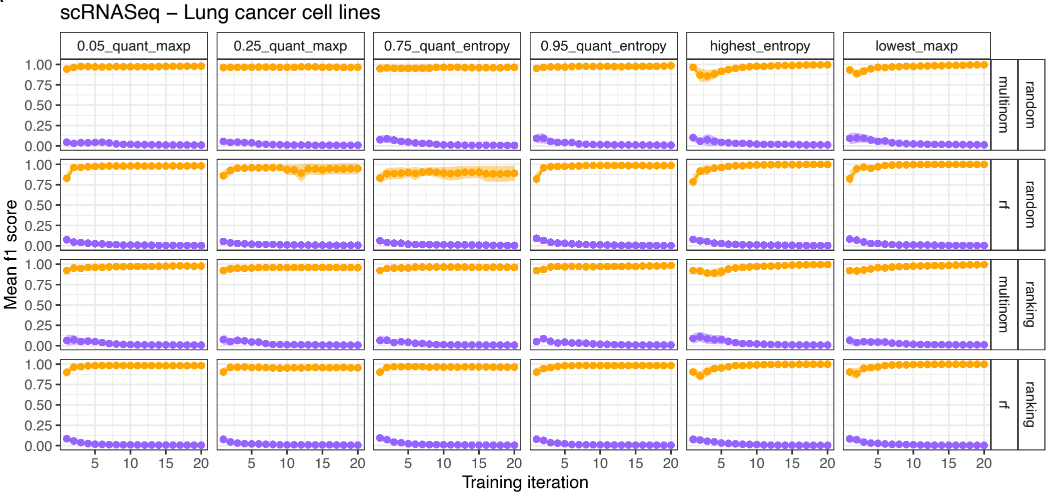

B

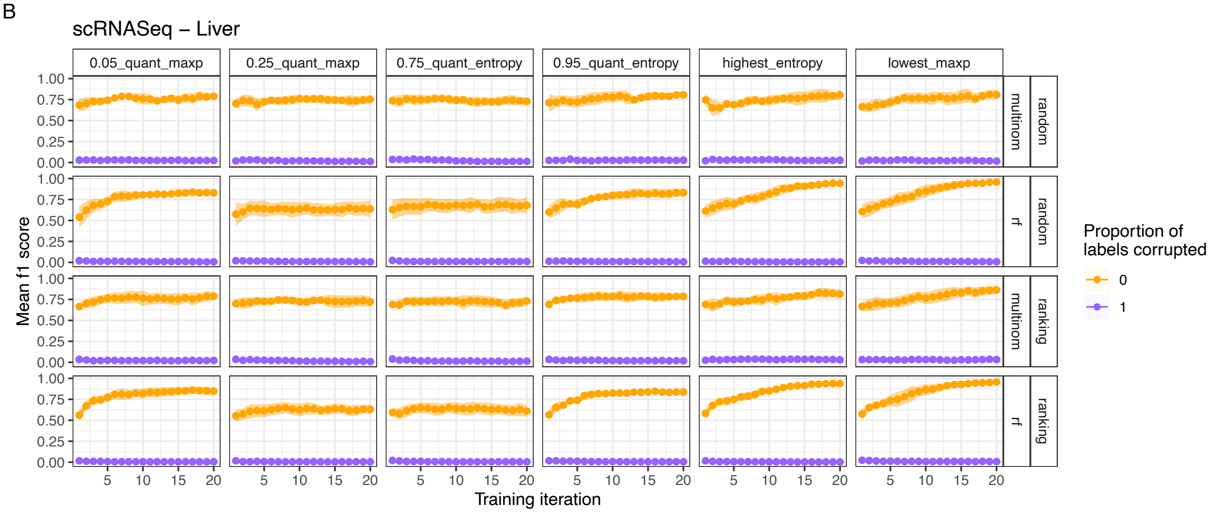

C

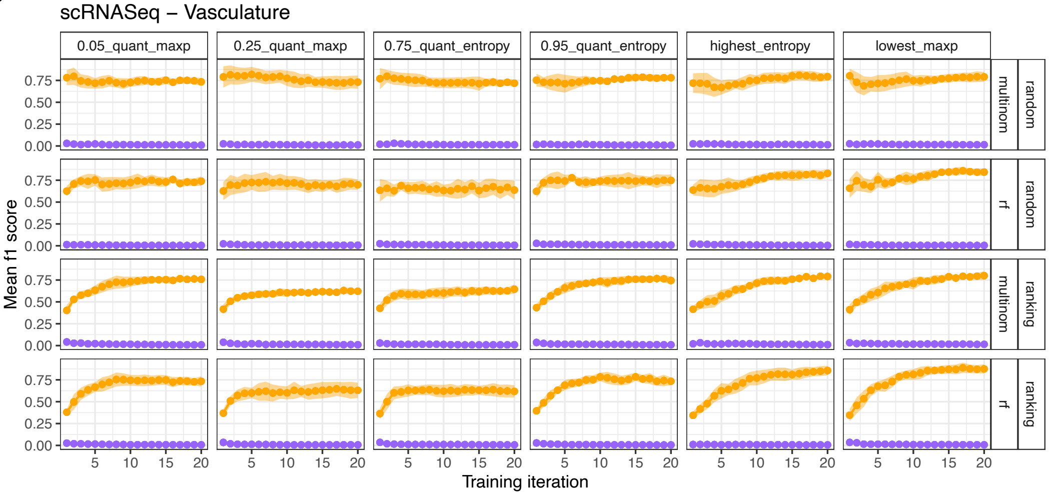

S. Figure 3. Active learning algorithms work as intended. Same as S. Figure 2 for A) scRNASeq - Lung cancer cell lines, B) scRNASeq - Liver and C) scRNASeq - Vasculature. Source data are provided on zenodo: https://doi.org/10.5281/zenodo.10403475.

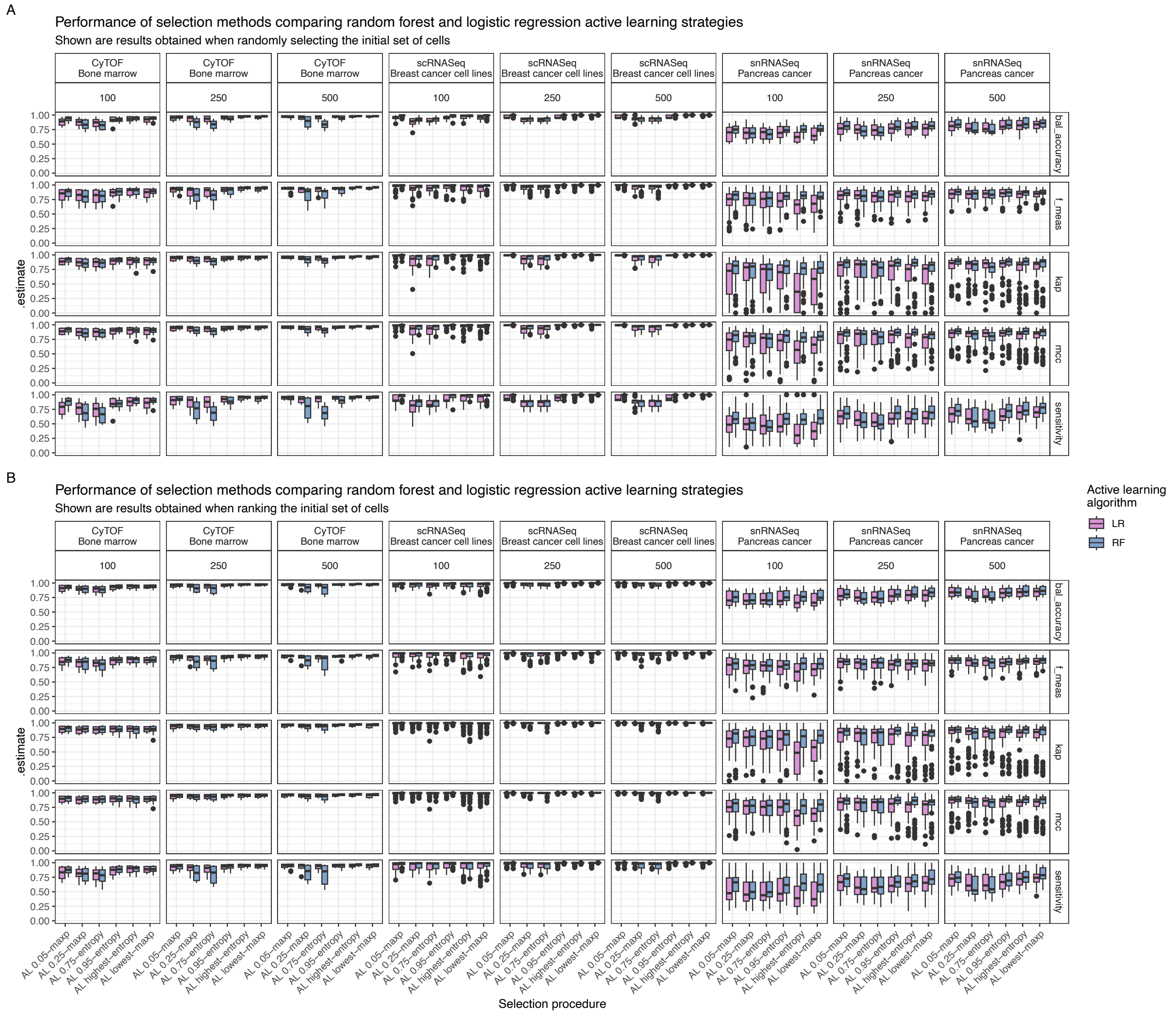

S. Figure 4. Performance of active learning methods by active learning model. Shown are the five accuracy measures across all ten train test splits for the CyTOF - Bone marrow, scRNASeq - Breast cancer cell lines and snRNASeq - Pancreas cancer cohorts and selected dataset size coloured by the active learning model used. A) Results when the initial set of cells were selected randomly. B) Results when the initial set of cells was selected by ranking their expression. Boxplots depict the median as the center line, the boxes define interquartile range (IQR), the whiskers extend up to 1.5 times the IQR and all points depict outliers from this range. Source data are provided on zenodo: https://doi.org/10.5281/zenodo.10403475.

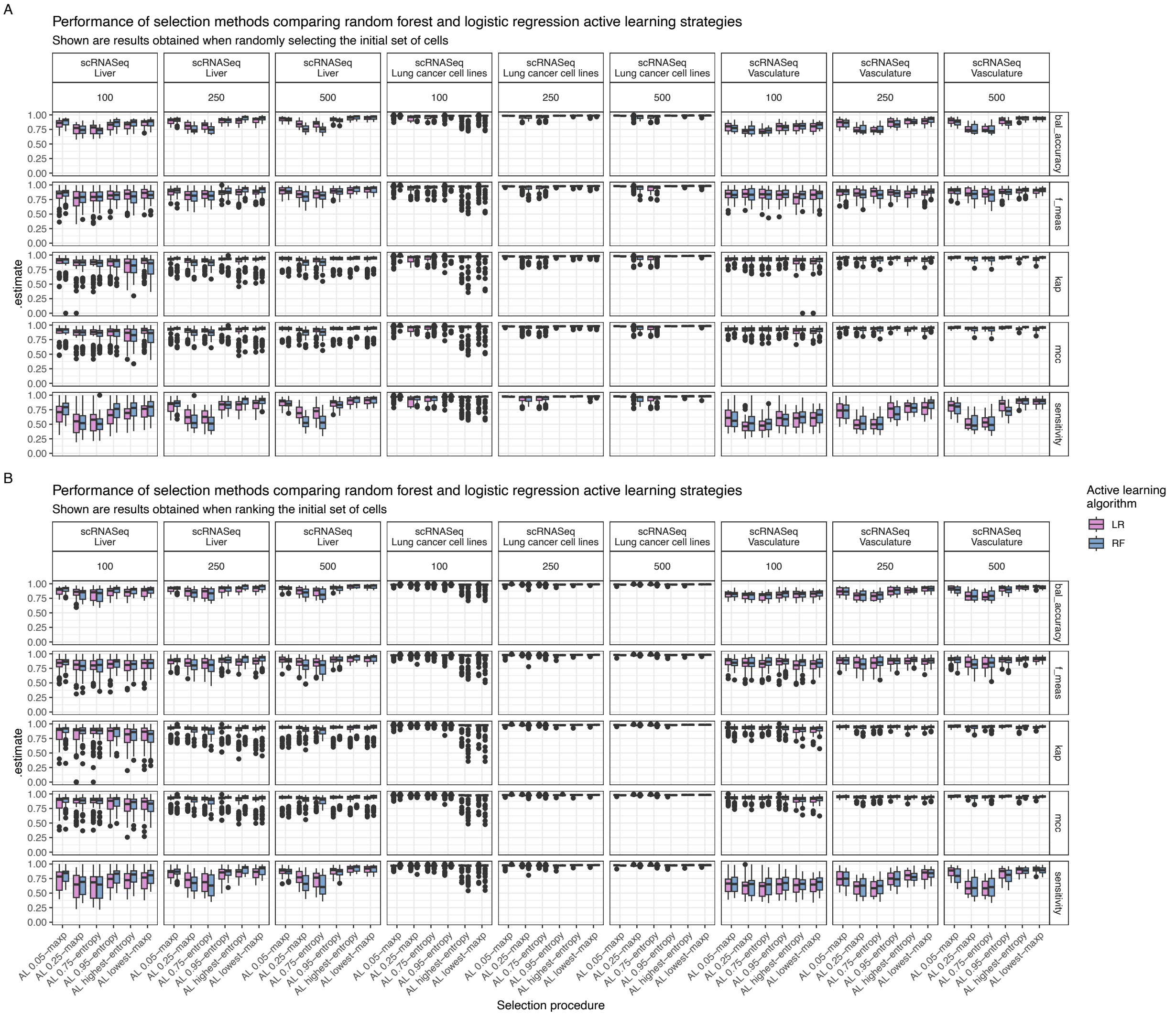

S. Figure 5. Performance of active learning methods by active learning model. Same as S. Figure 4 for the scRNASeq - Liver, scRNASeq - Lung cancer cell lines and scRNASeq - Vasculature datasets. A) Results when the initial set of cells were selected randomly. B) Results when the initial set of cells was selected by ranking their expression. Source data are provided on zenodo: https://doi.org/10.5281/zenodo.10403475.

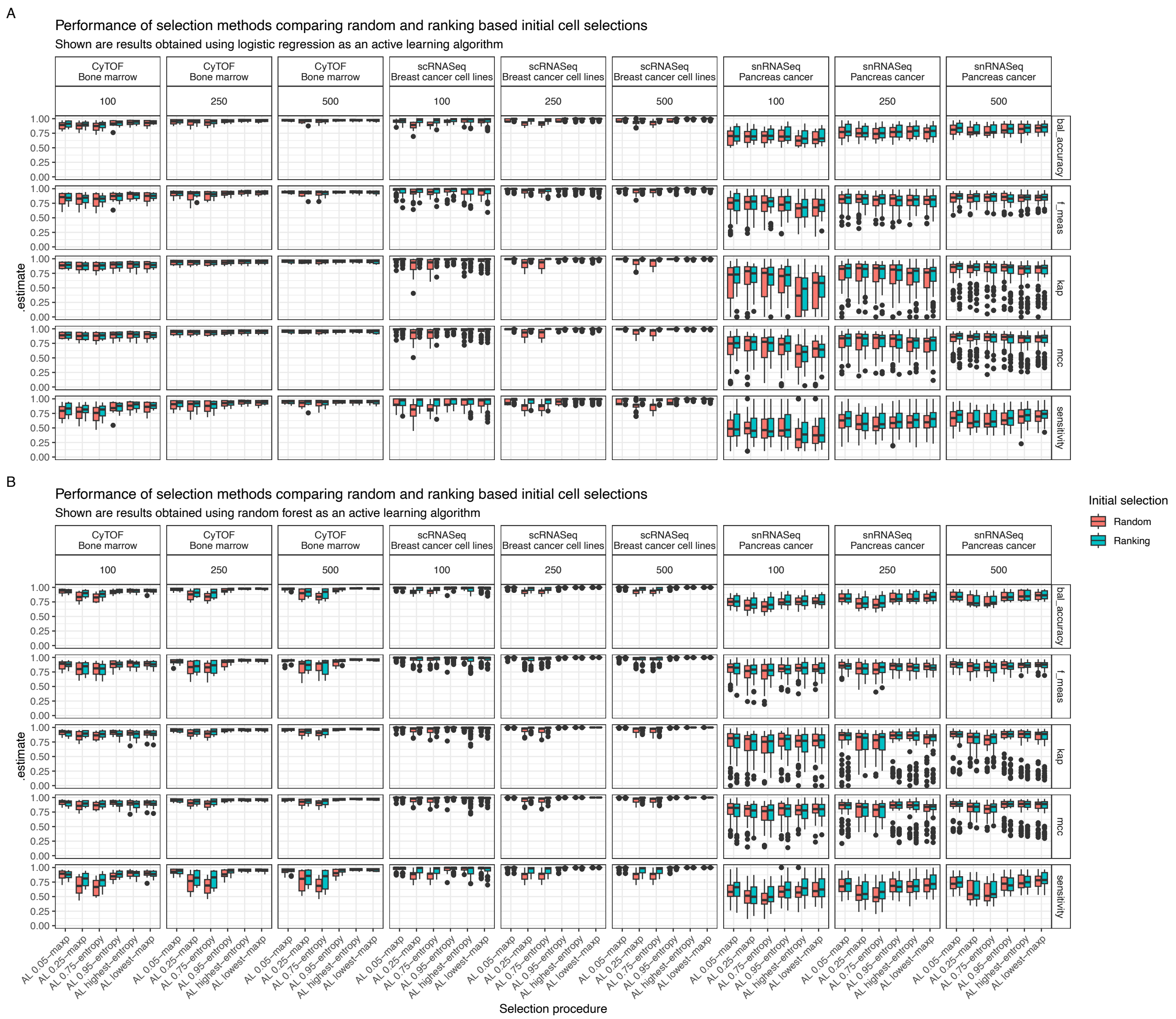

S. Figure 6. Performance of active learning methods by initial selection procedure. Shown are the five accuracy measures across all ten train test splits for the CyTOF - Bone marrow, scRNASeq - Breast cancer cell lines and snRNASeq - Pancreas cancer cohorts and selected dataset size coloured by the initial selection procedure. A) Results for the logistic regression model. B) Results for the random forest model. Boxplots depict the median as the center line, the boxes define interquartile range (IQR), the whiskers extend up to 1.5 times the IQR and all points depict outliers from this range. Source data are provided on zenodo: https://doi.org/10.5281/zenodo.10403475.

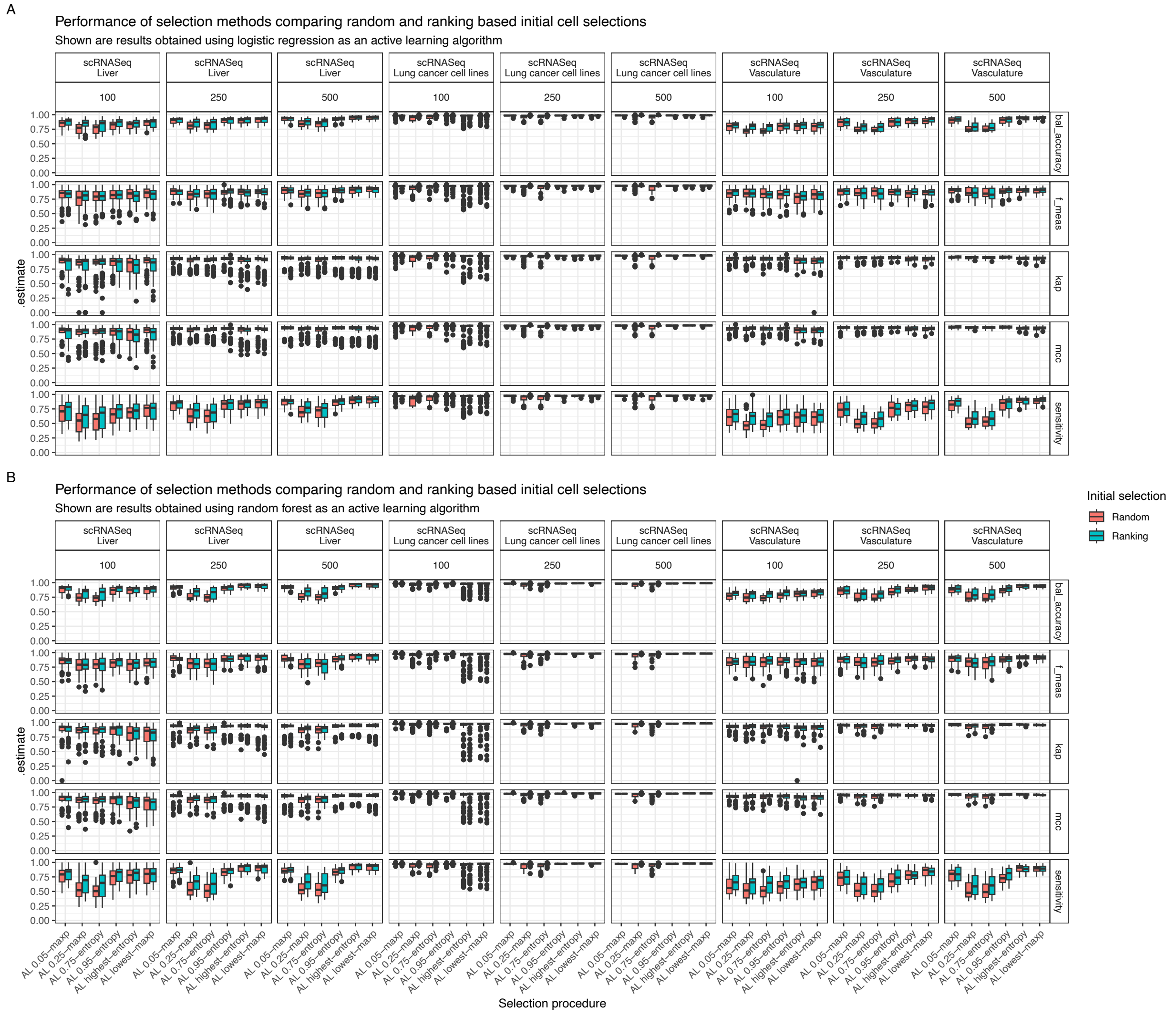

S. Figure 7. Performance of active learning methods by initial selection procedure. Same as S. Figure 6 for the scRNASeq - Liver, scRNASeq - Lung cancer cell lines and scRNASeq - Vasculature datasets. A) Results for the logistic regression model. B) Results for the random forest model. Source data are provided on zenodo: https://doi.org/10.5281/zenodo.10403475.

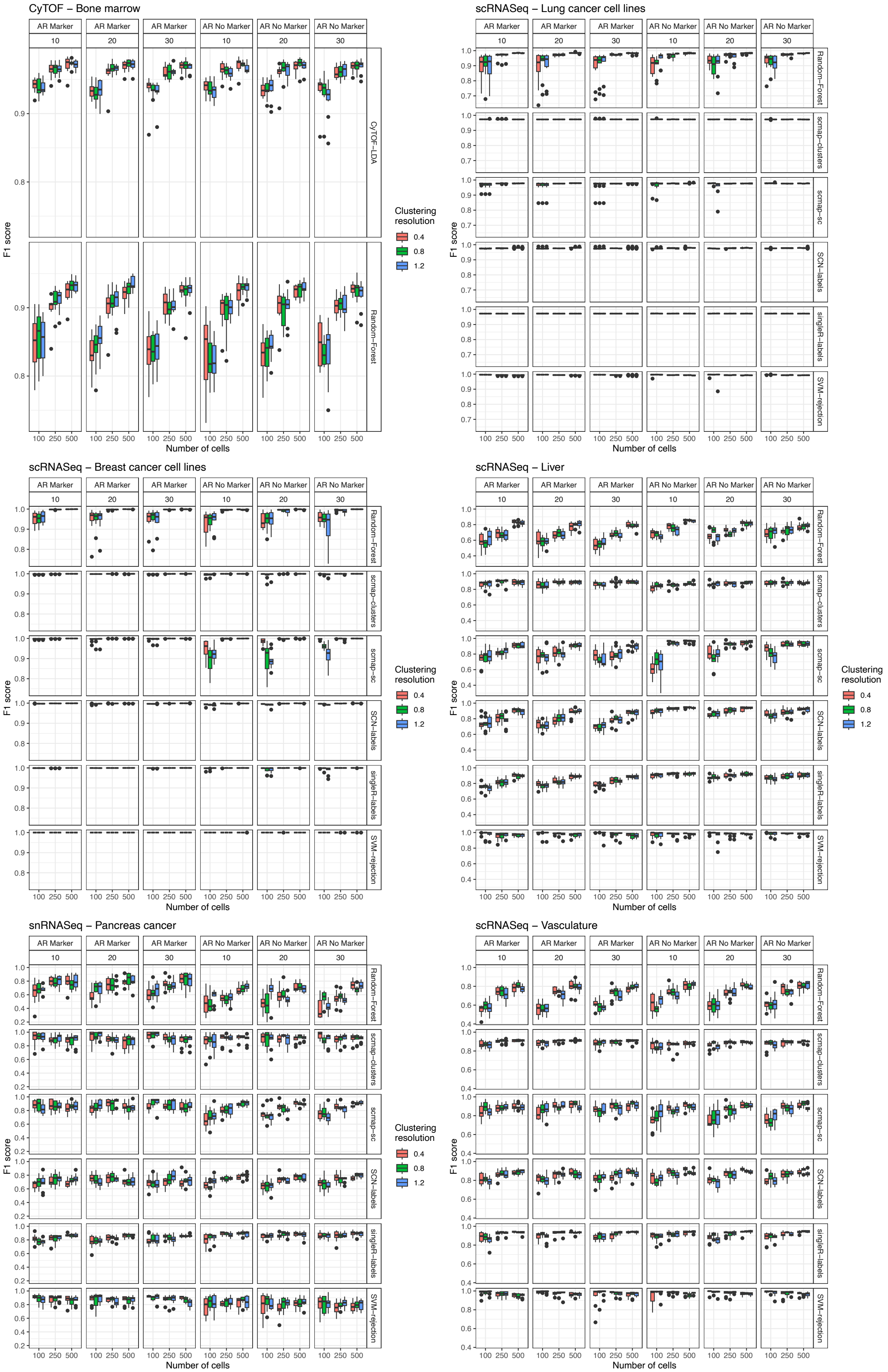

CyTOF – Bone marrow
scRNASeq – Lung cancer cell lines
scRNASeq – Breast cancer cell lines
scRNASeq – Liver
snRNASeq – Pancreas cancer
scRNASeq – Vasculature
AR Marker
AR No Marker
10
20
30
F1 score
Number of cells
100
250
500
Clustering resolution
0.4
0.8
1.2
CyTOF–LDA
Random–Forest
scmap–clusters
scmap–sc
SCN–labels
singleR–labels
SVM–rejection

S. Figure 8. Effect of clustering resolution on predictive performance of classifiers. Shown is the F1-score for the marker aware and unaware adaptive reweighting methods across all ten train test splits faceted by a different number of k-nearest neighbours and cell type prediction methods. Boxplots depict the median as the center line, the boxes define interquartile range (IQR), the whiskers extend up to 1.5 times the IQR and all points depict outliers from this range. Source data are provided on zenodo: https://doi.org/10.5281/zenodo.10403475.

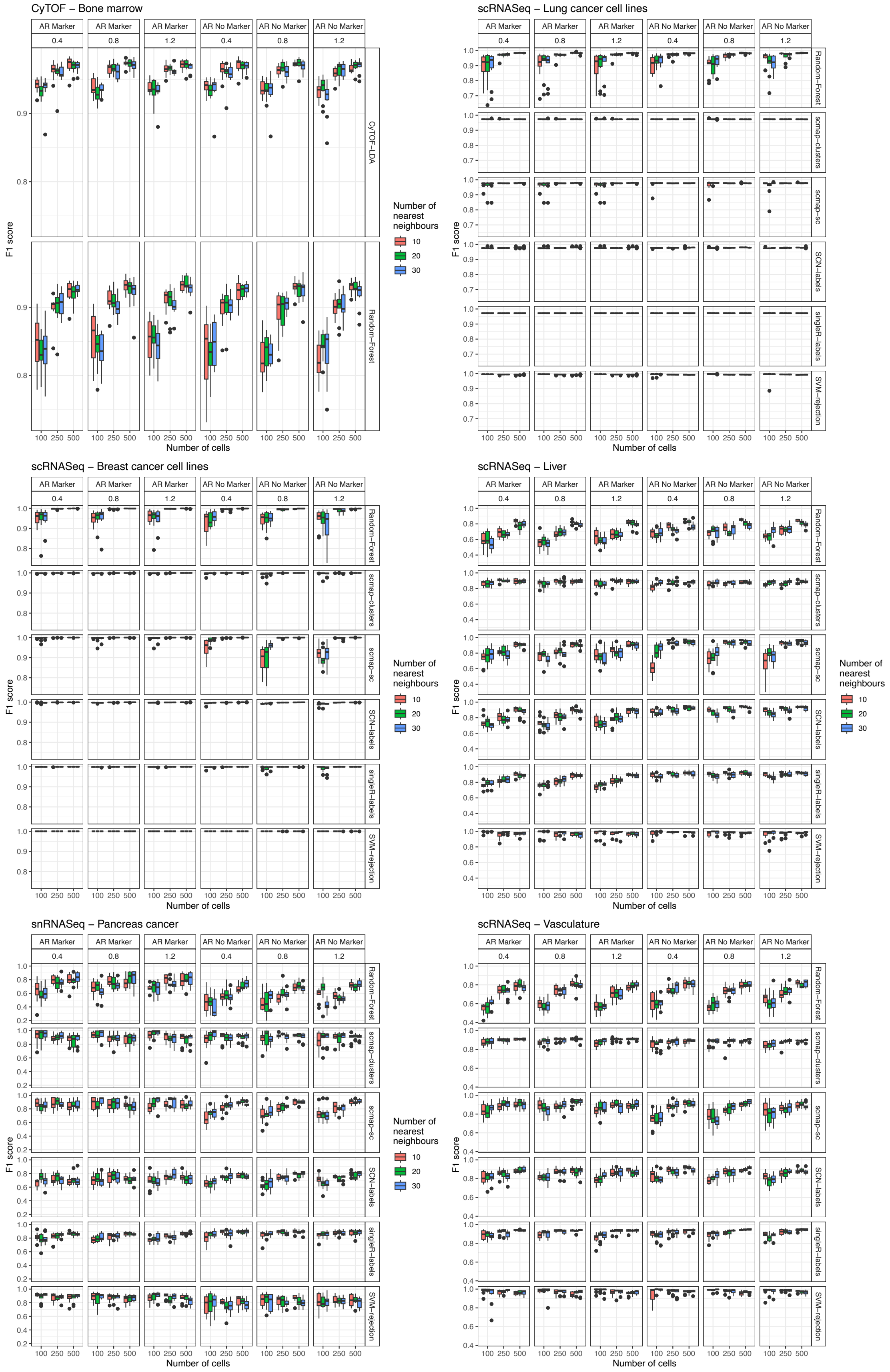

CyTOF – Bone marrow
scRNASeq – Lung cancer cell lines
scRNASeq – Breast cancer cell lines
scRNASeq – Liver
snRNASeq – Pancreas cancer
scRNASeq – Vasculature
AR Marker
AR No Marker
0.4
0.8
1.2
CyTOF–LDA
Random–Forest
scmap–clusters
scmap–sc
SCN–labels
singleR–labels
SVM–rejection
F1 score
Number of cells
100 250 500
Number of nearest neighbours
10
20
30

S. Figure 9. Effect of k nearest neighbor parameter on predictive performance of classifiers. Shown is the F1-score for the marker aware and unaware adaptive reweighting methods across all ten train test splits faceted by the clustering resolution and cell type prediction methods. Boxplots depict the median as the center line, the boxes define interquartile range (IQR), the whiskers extend up to 1.5 times the IQR and all points depict outliers from this range. Source data are provided on zenodo: https://doi.org/10.5281/zenodo.10403475.

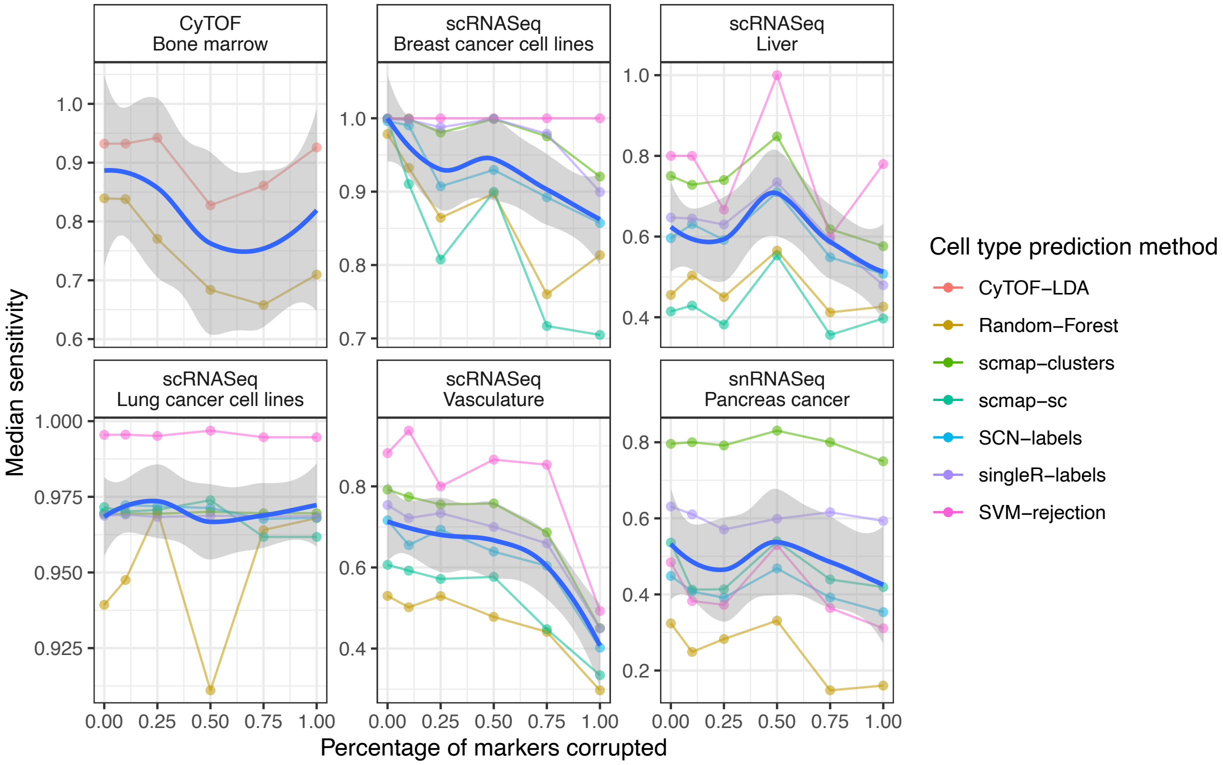

S. Figure 10. Effect of adaptive reweighting marker corruption on sensitivity. Shown is the median sensitivity across all ten train test splits for each method and dataset as the markers used to select the initial cell population are increasingly corrupted from 0 to 100%. Source data are provided on zenodo: https://doi.org/10.5281/zenodo.10403475.

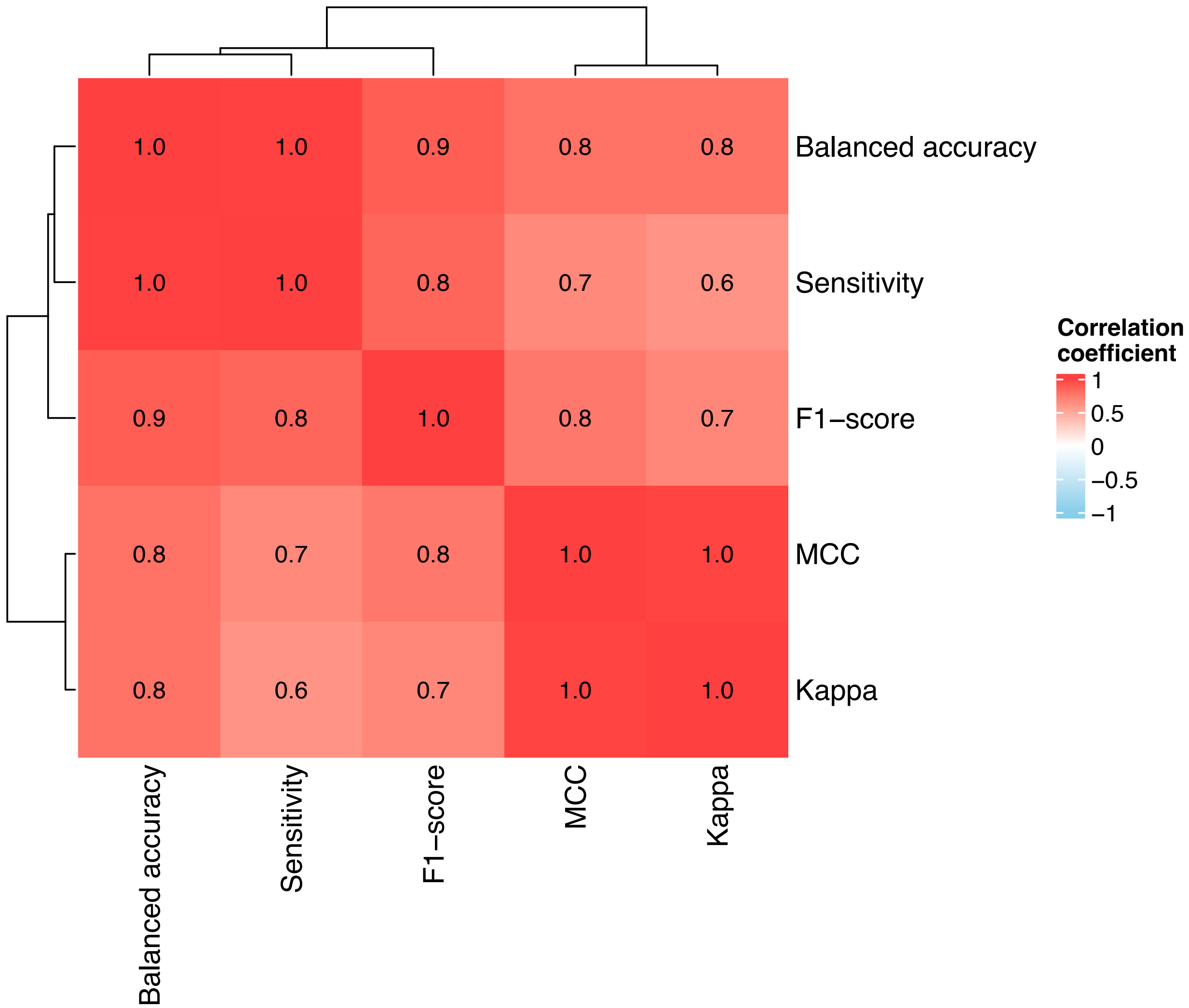

S. Figure 11. Performance metrics correlate highly with each other. Shown is the pearson correlation coefficient between all metrics. The correlated values are the average performance across the ten seeds. Source data are provided on zenodo: https://doi.org/10.5281/zenodo.10403475.

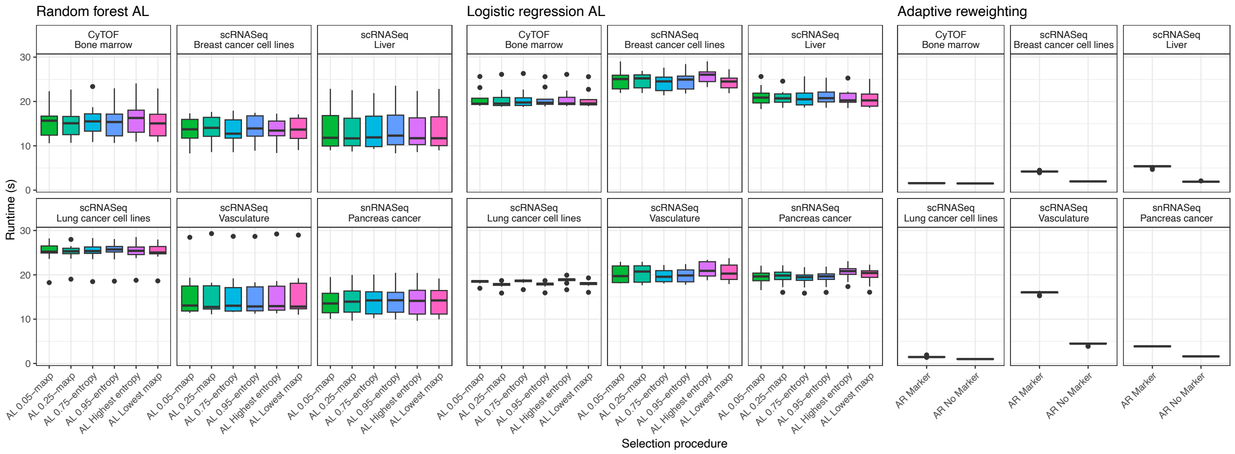

S. Figure 12. Runtime analysis for all selection methods benchmarked. Shown is the runtime in seconds for each selection method and dataset for each of the ten train test splits. All active learning methods were trained using a random set of 20 initial cells. Boxplots depict the median as the center line, the boxes define interquartile range (IQR), the whiskers extend up to 1.5 times the IQR and all points depict outliers from this range. Source data are provided on zenodo: https://doi.org/10.5281/zenodo.10403475.

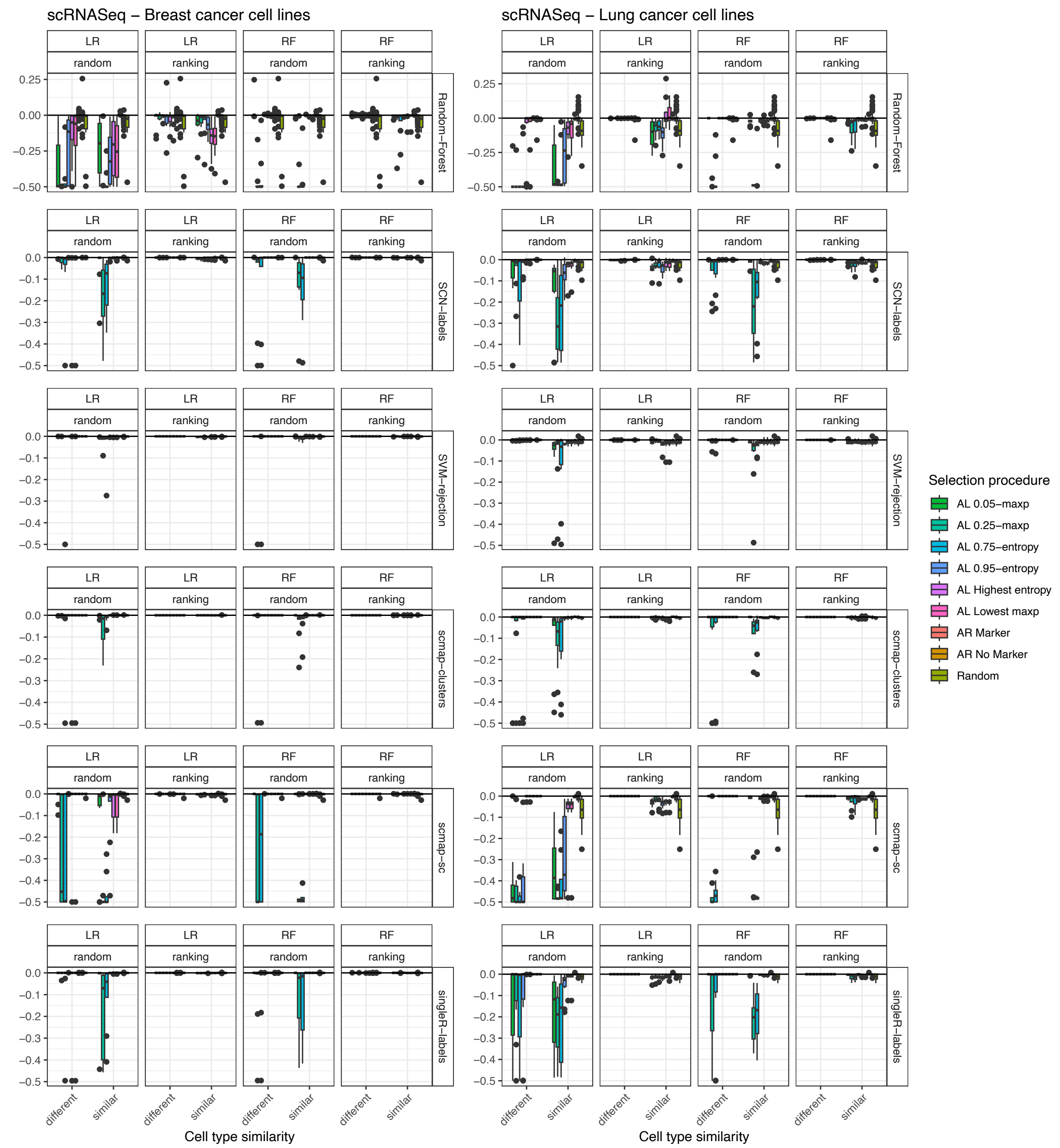

S. Figure 13. Effect of dataset imbalance on balanced accuracy. Shown is the change in balanced accuracy (calculated as accuracy in imbalanced dataset - accuracy in balanced dataset / accuracy in balanced dataset). Each figure is faceted by the active learning model used (LR or RF), the cell selection method for the first 20 cells and the cell type prediction method. Boxplots depict the median as the center line, the boxes define interquartile range (IQR), the whiskers extend up to 1.5 times the IQR and all points depict outliers from this range. Source data are provided on zenodo: https://doi.org/10.5281/zenodo.10403475.

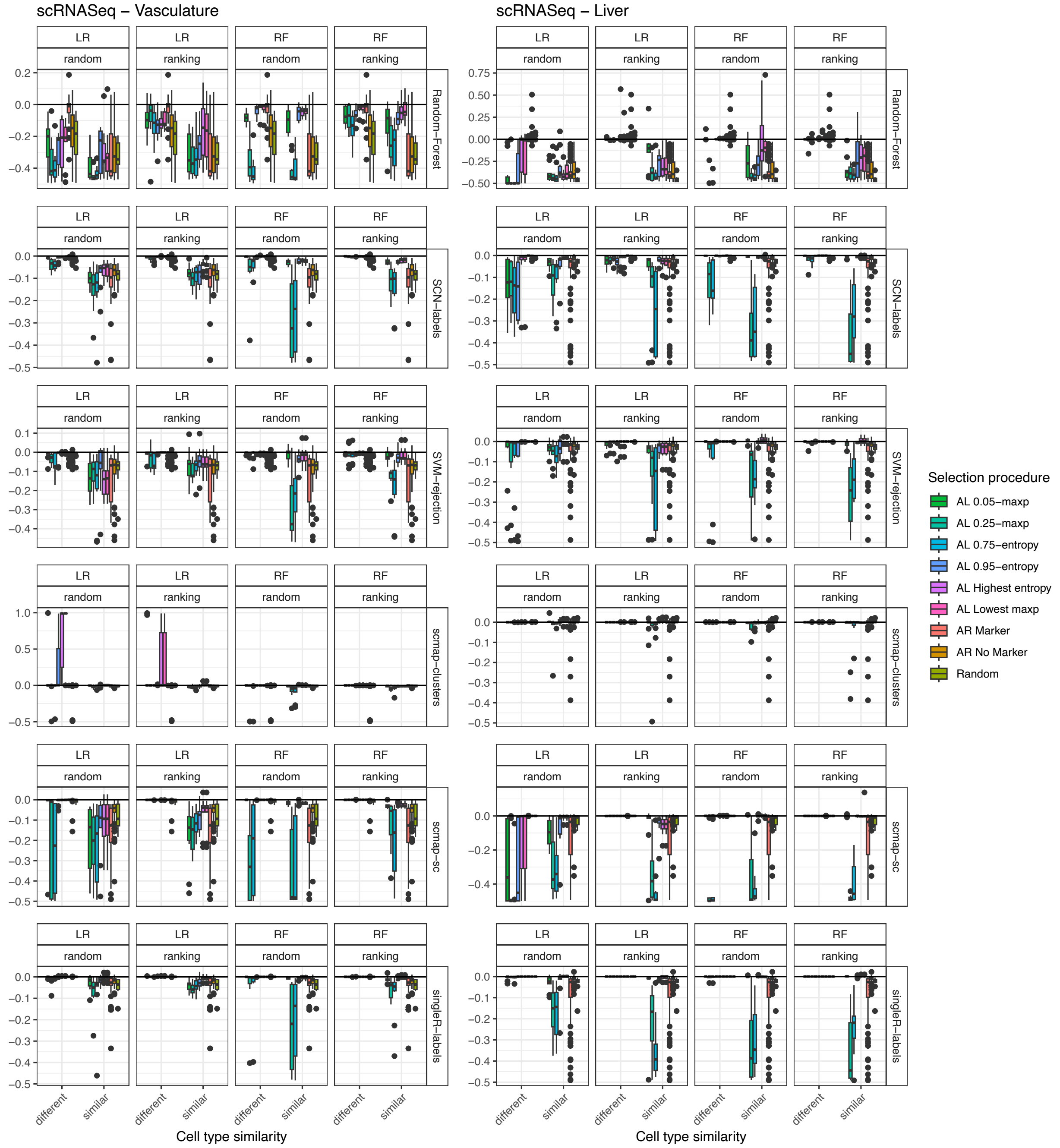

S. Figure 14. Effect of dataset imbalance on classification accuracy. Same as S. Figure 13 for the tabula vasculature and liver atlas datasets. Source data are provided on zenodo: https://doi.org/10.5281/zenodo.10403475.

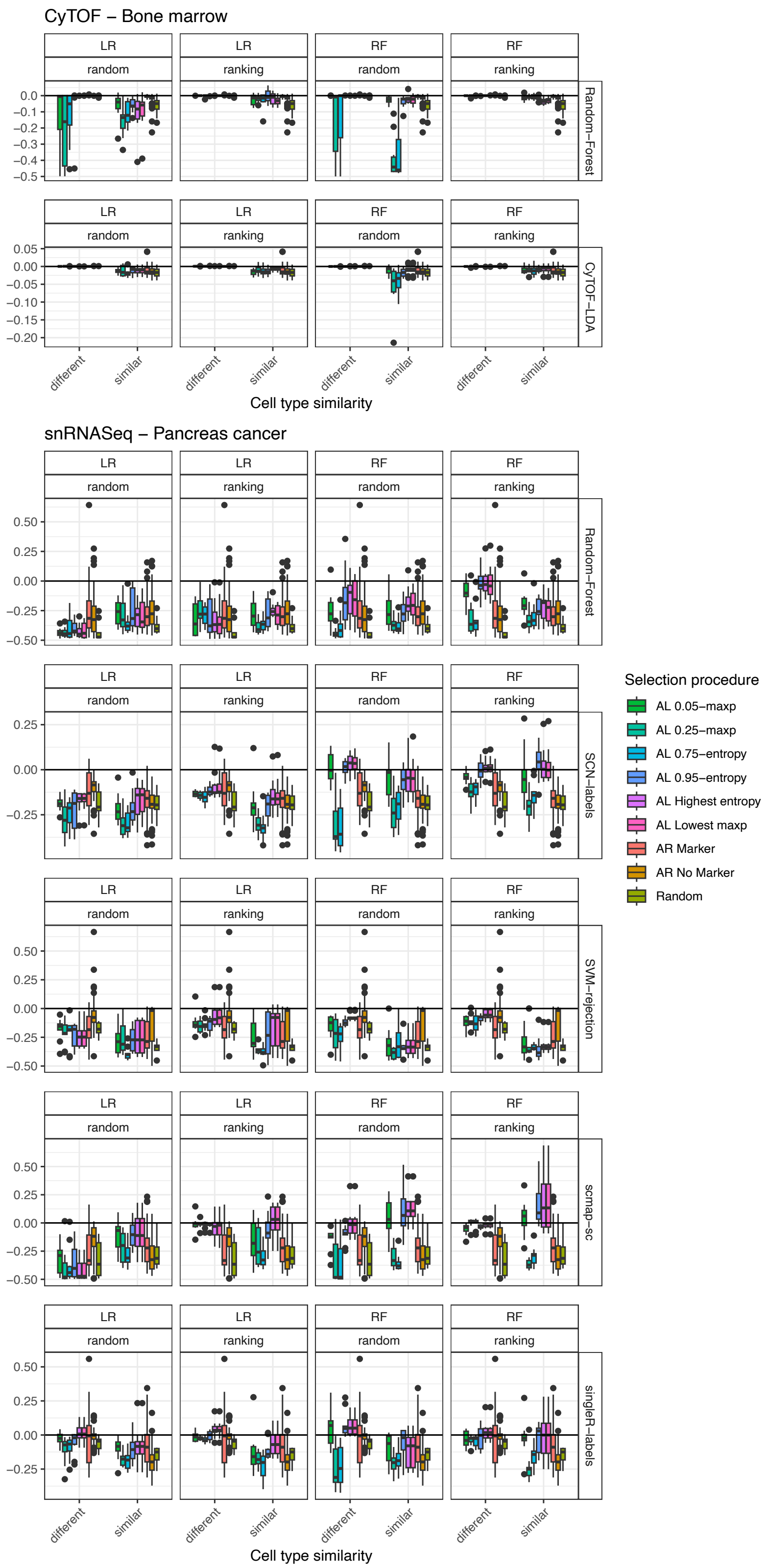

CyTOF – Bone marrow
LR
RF
random
ranking
Random–Forest
CyTOF–LDA
different
similar
Cell type similarity
snRNASeq – Pancreas cancer
SCN–labels
SVM–rejection
scmap–sc
singleR–labels
Selection procedure
AL 0.05–maxp
AL 0.25–maxp
AL 0.75–entropy
AL 0.95–entropy
AL Highest entropy
AL Lowest maxp
AR Marker
AR No Marker
Random

S. Figure 15. Effect of dataset imbalance on classification accuracy. Same as S. Figure 13 for the CyTOF bone marrow and scRNASeq lung cancer cell line dataset. Source data are provided on zenodo: https://doi.org/10.5281/zenodo.10403475.

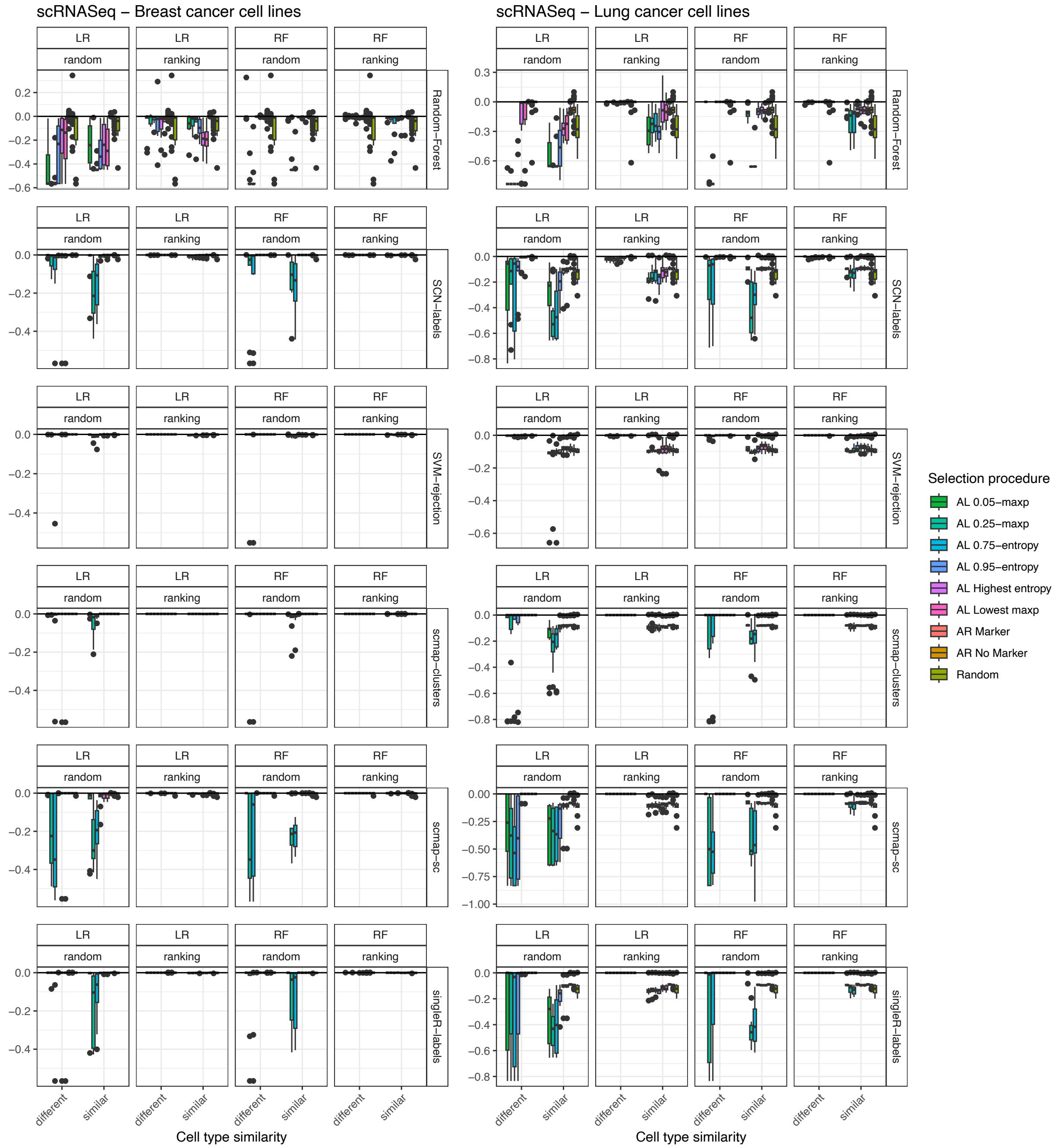

S. Figure 16. Effect of dataset imbalance on F1-score. Shown is the change in F1-score (calculated as F1-score in imbalanced dataset - F1-score in balanced dataset / F1-score in balanced dataset). Each figure is faceted by the active learning model used (LR or RF), the cell selection method for the first 20 cells and the cell type prediction method. Boxplots depict the median as the center line, the boxes define interquartile range (IQR), the whiskers extend up to

1.5 times the IQR and all points depict outliers from this range. Source data are provided on zenodo: https://doi.org/10.5281/zenodo.10403475.

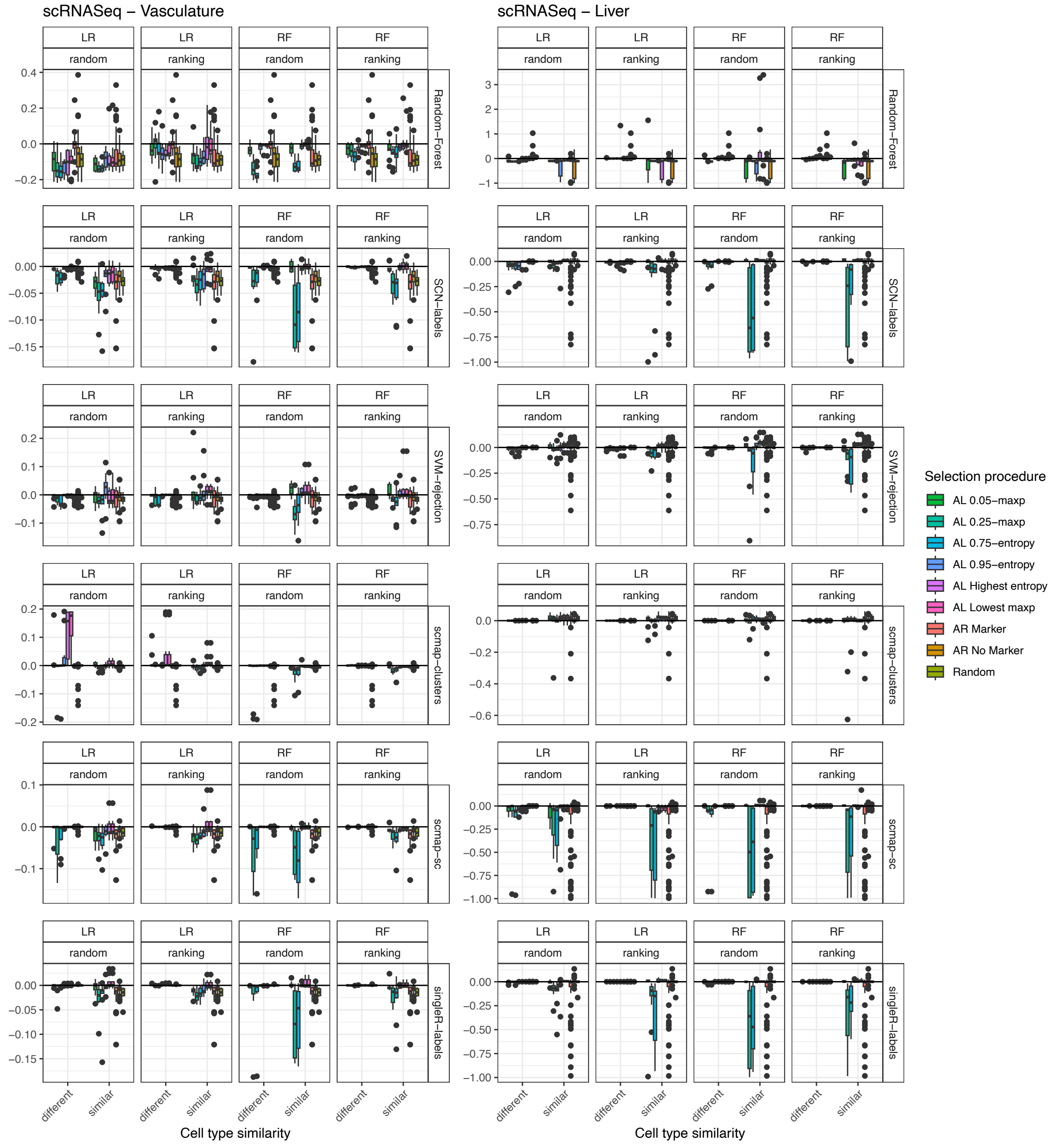

S. Figure 17. Effect of dataset imbalance on F1-score. Same as S. Figure 16 for the tabula vasculature and liver atlas datasets. Source data are provided on zenodo: https://doi.org/10.5281/zenodo.10403475.

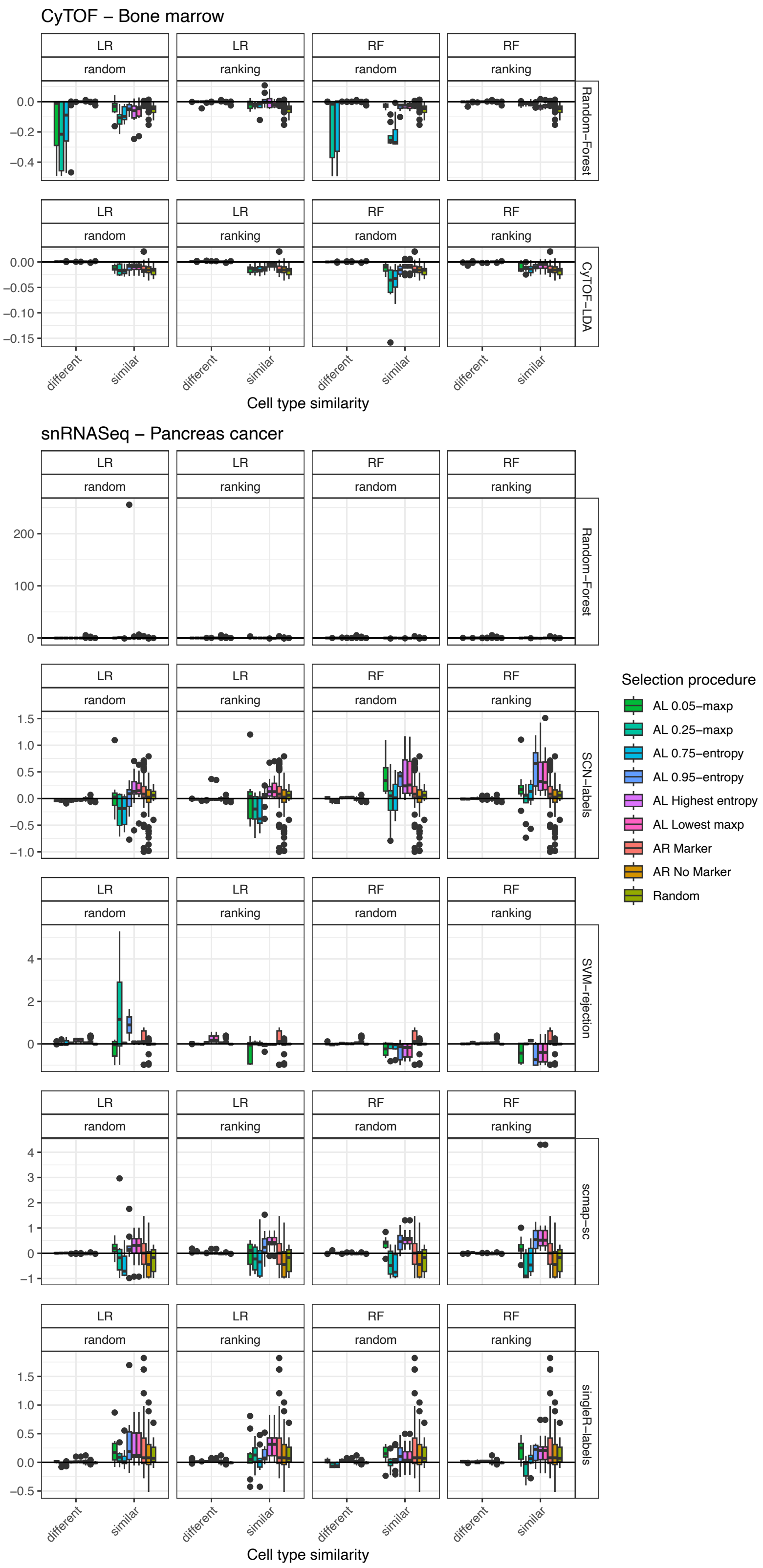
CyTOF – Bone marrow
LR
RF
random
ranking
Random–Forest
CyTOF–LDA
0.0
−0.2
−0.4
0.00
−0.05
−0.10
−0.15
different
similar
Cell type similarity
snRNASeq – Pancreas cancer
Random–Forest
SCN–labels
SVM–rejection
scmap–sc
singleR–labels
200
100
0
1.5
1.0
0.5
0.0
−0.5
−1.0
4
2
3
1
−1
Selection procedure
AL 0.05–maxp
AL 0.25–maxp
AL 0.75–entropy
AL 0.95–entropy
AL Highest entropy
AL Lowest maxp
AR Marker
AR No Marker
Random

S. Figure 18. Effect of dataset imbalance on F1-score. Same as S. Figure 16 for the CyTOF bone marrow and scRNASeq lung cancer cell line dataset. Source data are provided on zenodo: https://doi.org/10.5281/zenodo.10403475.

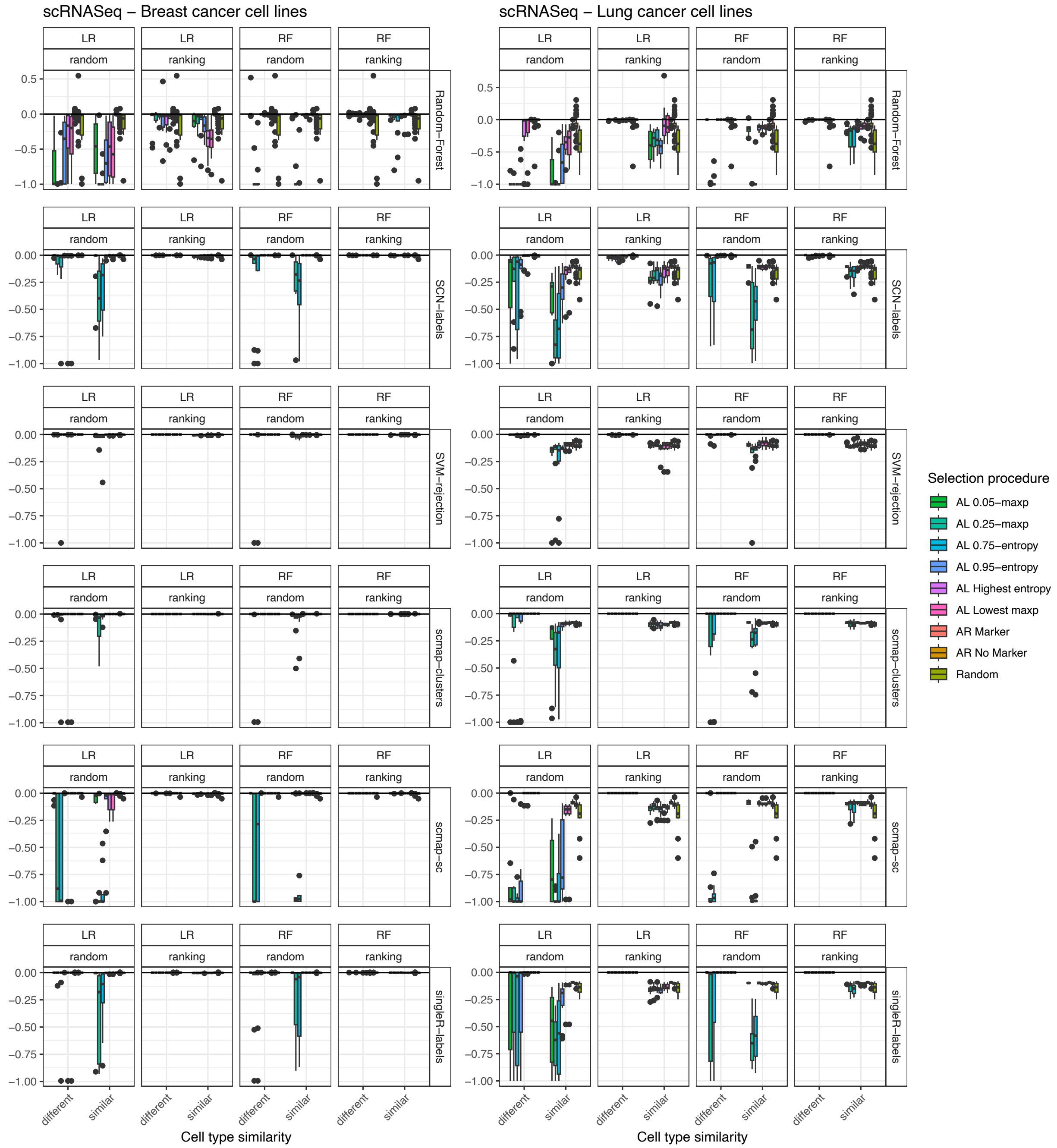

S. Figure 19. Effect of dataset imbalance on kappa. Shown is the change in kappa (calculated as kappa in imbalanced dataset - kappa in balanced dataset / kappa in balanced dataset). Each figure is faceted by the active learning model used (LR or RF), the cell selection method for the first 20 cells and the cell type prediction method. Boxplots depict the median as

the center line, the boxes define interquartile range (IQR), the whiskers extend up to 1.5 times the IQR and all points depict outliers from this range. Source data are provided on zenodo: https://doi.org/10.5281/zenodo.10403475.

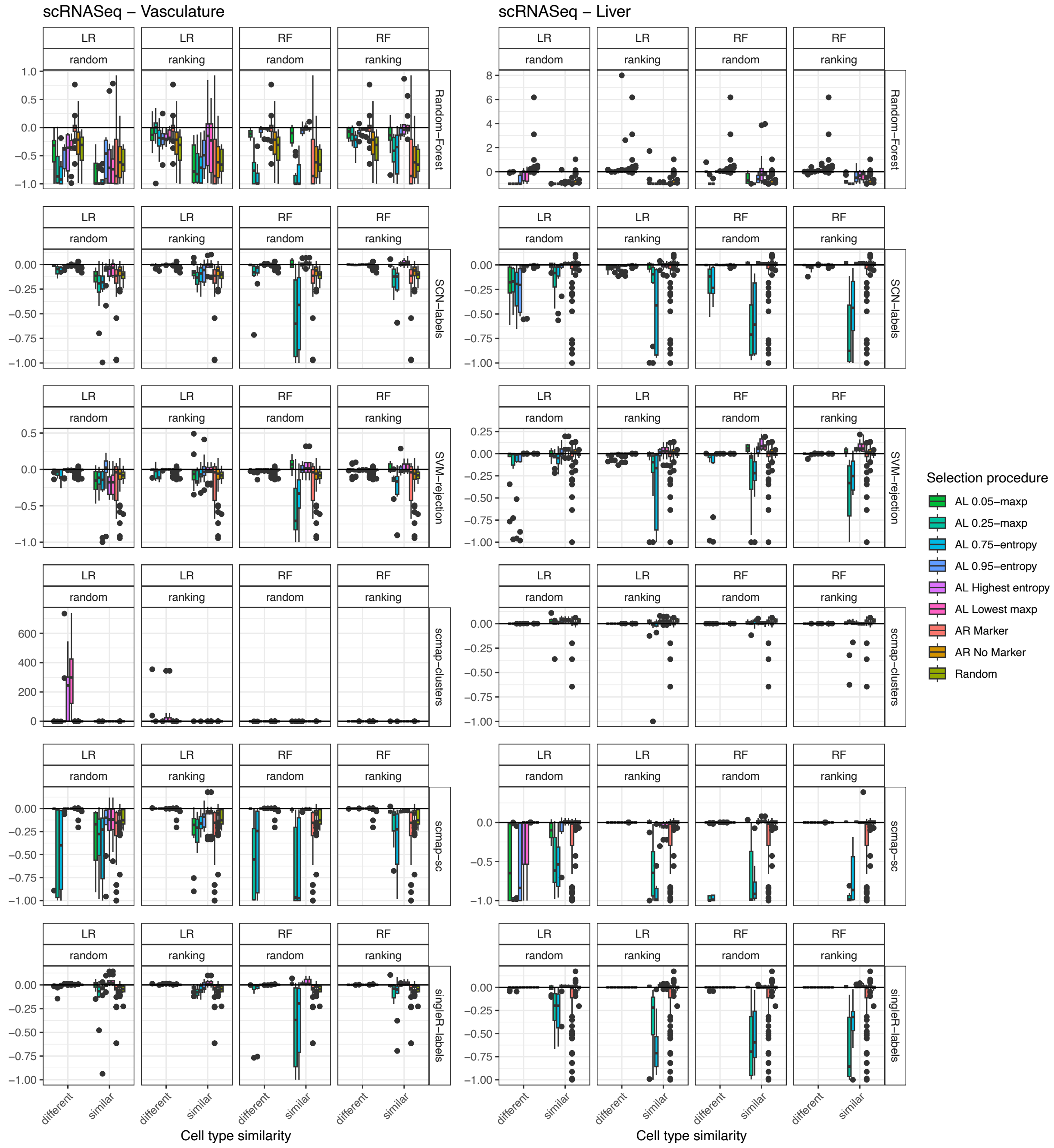

S. Figure 20. Effect of dataset imbalance on kappa. Same as S. Figure 19 for the tabula vasculature and liver atlas datasets. Source data are provided on zenodo: https://doi.org/10.5281/zenodo.10403475.

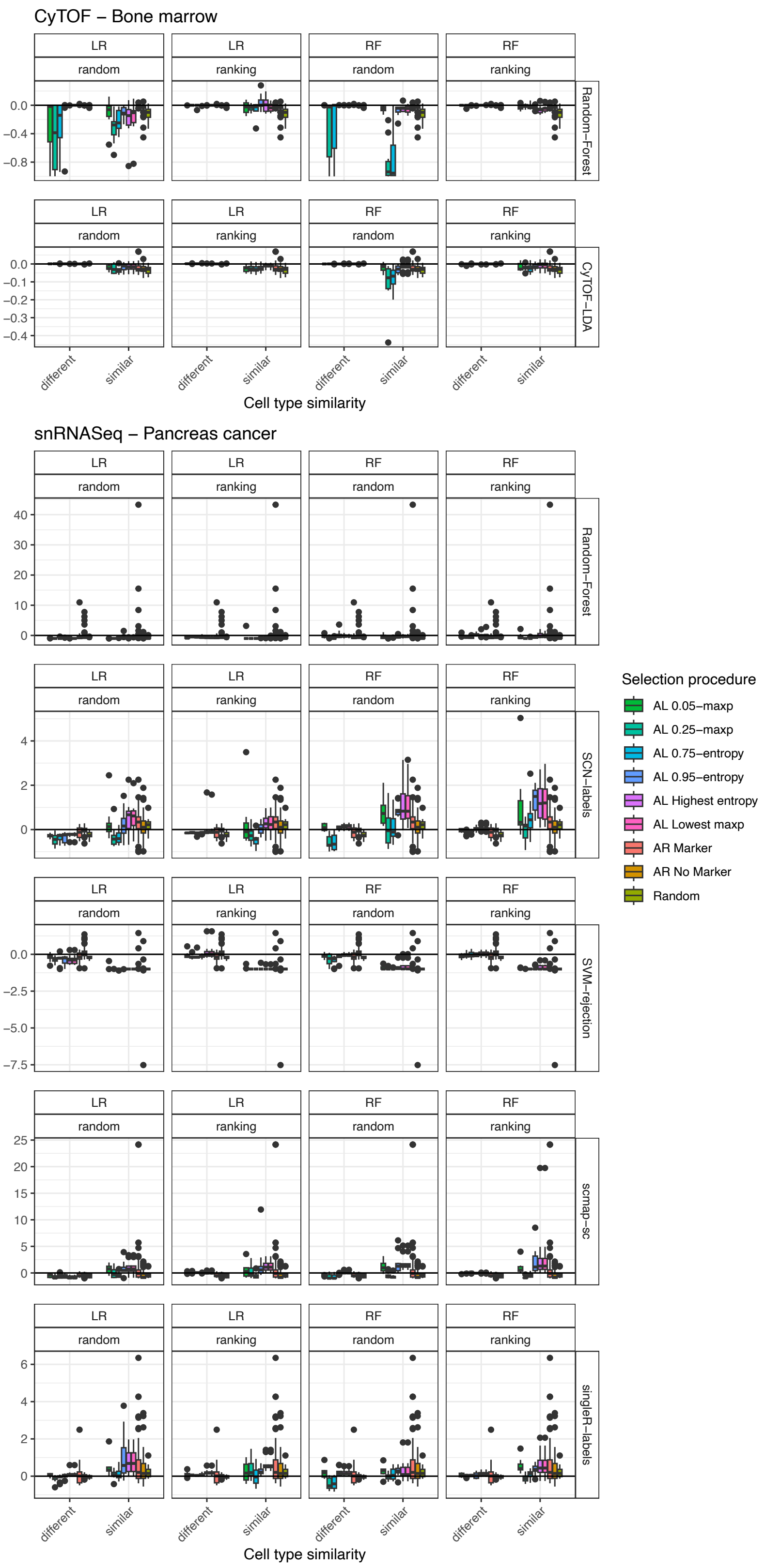

CyTOF – Bone marrow
LR
RF
random
ranking
Random–Forest
CyTOF–LDA
Cell type similarity
different
similar
snRNASeq – Pancreas cancer
SCN–labels
SVM–rejection
scmap–sc
singleR–labels
Selection procedure
AL 0.05–maxp
AL 0.25–maxp
AL 0.75–entropy
AL 0.95–entropy
AL Highest entropy
AL Lowest maxp
AR Marker
AR No Marker
Random

S. Figure 21. Effect of dataset imbalance on kappa. Same as S. Figure 19 for the CyTOF bone marrow and scRNASeq lung cancer cell line dataset. Source data are provided on zenodo: https://doi.org/10.5281/zenodo.10403475.

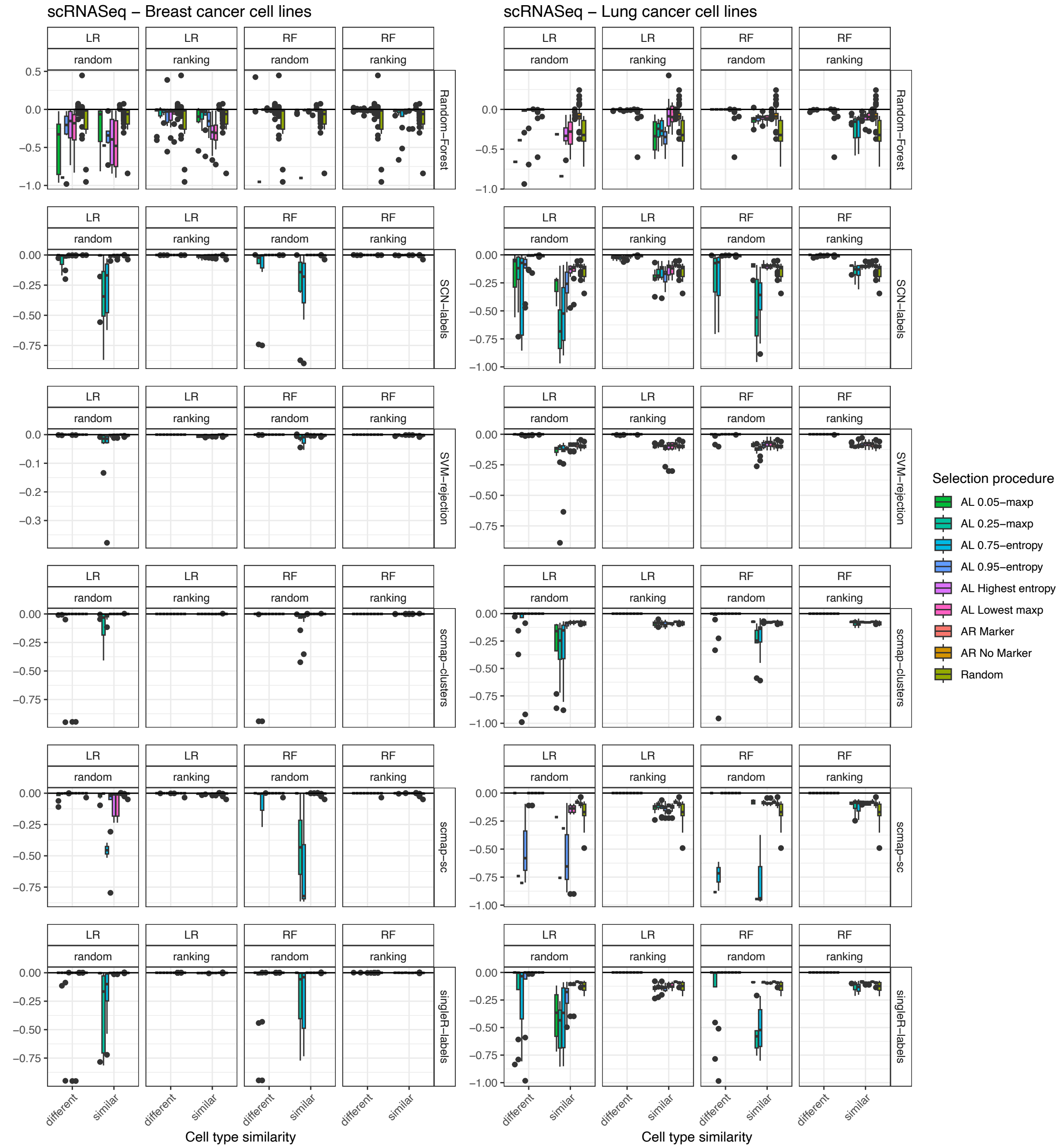

S. Figure 22. Effect of dataset imbalance on Matthew's correlation coefficient (MCC). Shown is the change in MCC (calculated as MCC in imbalanced dataset - MCC in balanced dataset / MCC in balanced dataset). Each figure is faceted by the active learning model used (LR or RF), the cell selection method for the first 20 cells and the cell type prediction method. Boxplots depict the median as the center line, the boxes define interquartile range (IQR), the

whiskers extend up to 1.5 times the IQR and all points depict outliers from this range. Source data are provided on zenodo: https://doi.org/10.5281/zenodo.10403475.

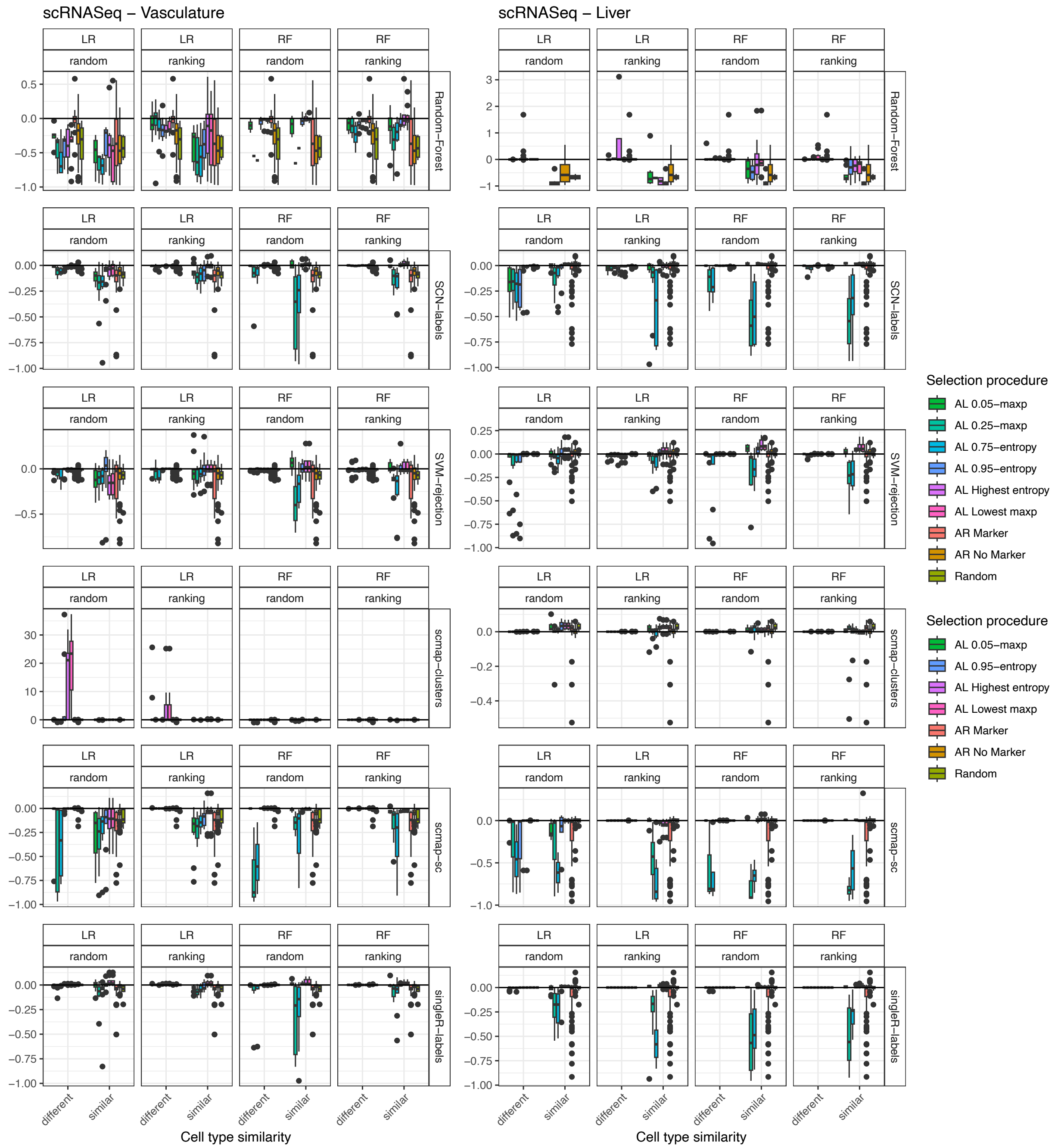

S. Figure 23. Effect of dataset imbalance on MCC. Same as S. Figure 22 for the tabula vasculature and liver atlas datasets. Source data are provided on zenodo: https://doi.org/10.5281/zenodo.10403475.

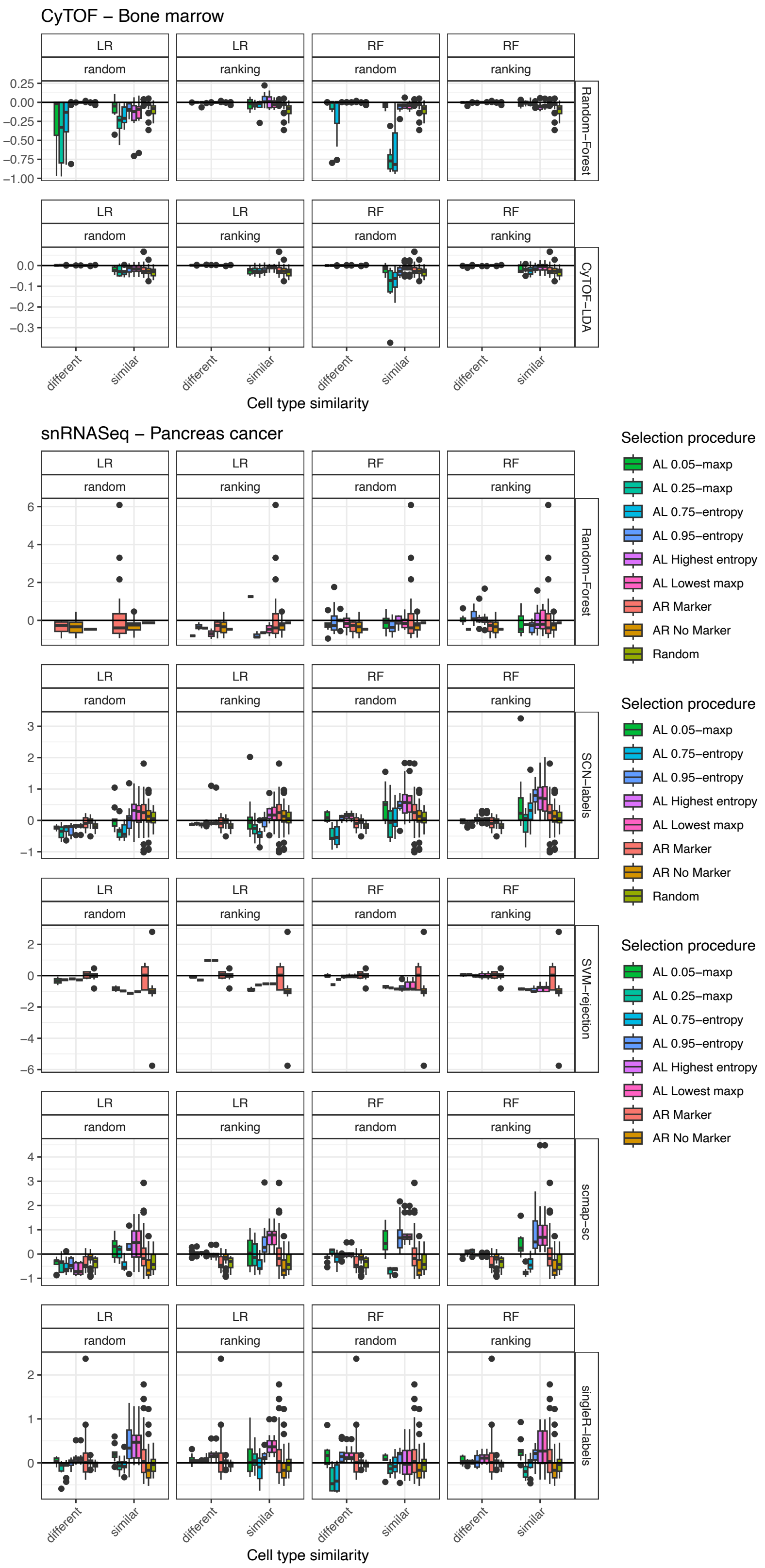

CyTOF – Bone marrow
LR
RF
random
ranking
Random–Forest
CyTOF–LDA
different
similar
Cell type similarity
snRNASeq – Pancreas cancer
Random–Forest
SCN–labels
SVM–rejection
scmap–sc
singleR–labels
Selection procedure
AL 0.05–maxp
AL 0.25–maxp
AL 0.75–entropy
AL 0.95–entropy
AL Highest entropy
AL Lowest maxp
AR Marker
AR No Marker
Random

S. Figure 24. Effect of dataset imbalance on MCC. Same as S. Figure 22 for the CyTOF bone marrow and scRNASeq lung cancer cell line dataset. Source data are provided on zenodo: https://doi.org/10.5281/zenodo.10403475.

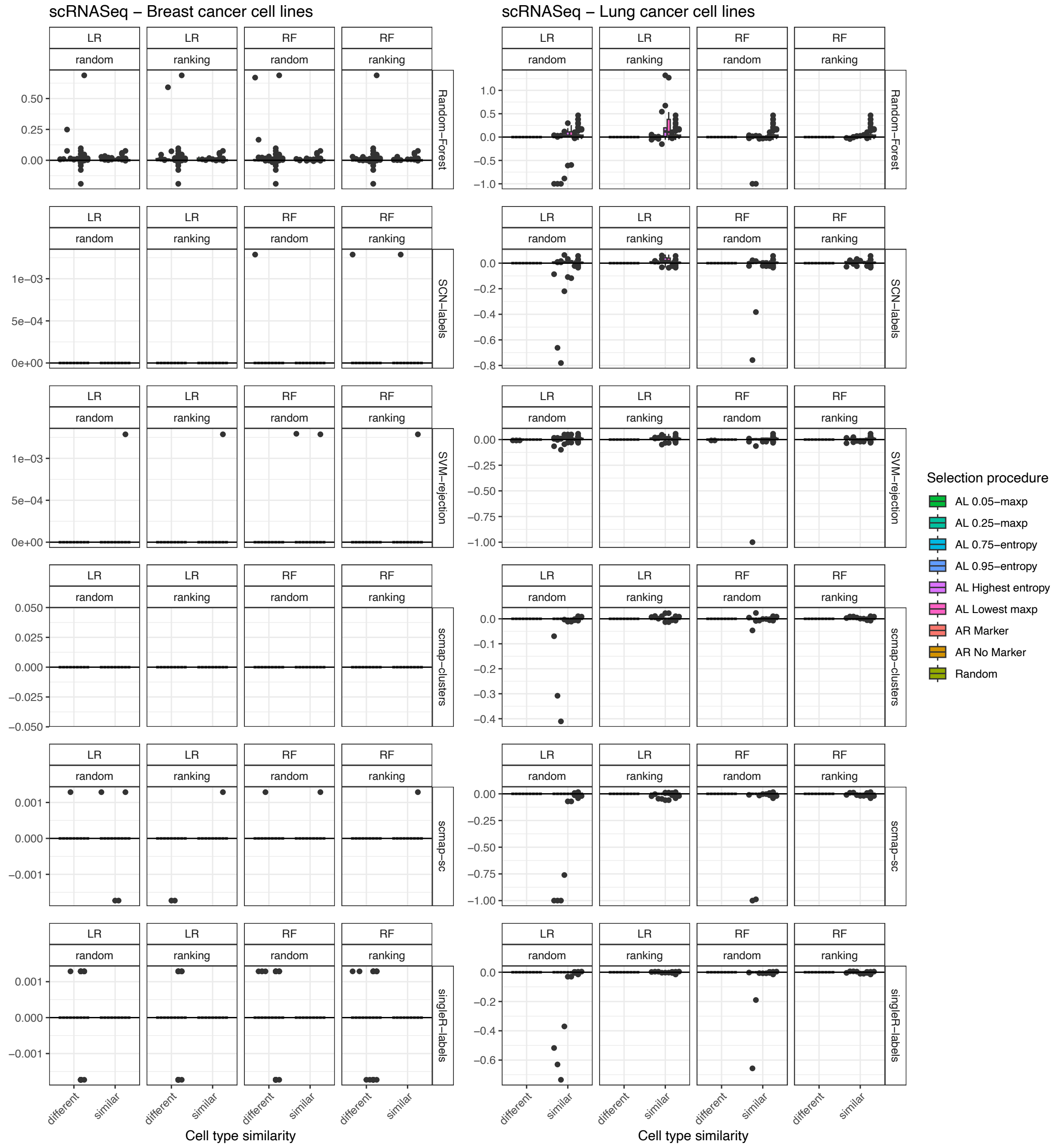

S. Figure 25. Effect of dataset imbalance on sensitivity. Shown is the change in MCC (calculated as sensitivity in imbalanced dataset - sensitivity in balanced dataset / sensitivity in balanced dataset). Each figure is faceted by the active learning model used (LR or RF), the cell selection method for the first 20 cells and the cell type prediction method. Boxplots depict the median as the center line, the boxes define interquartile range (IQR), the whiskers extend up to

1.5 times the IQR and all points depict outliers from this range. Source data are provided on zenodo: https://doi.org/10.5281/zenodo.10403475.

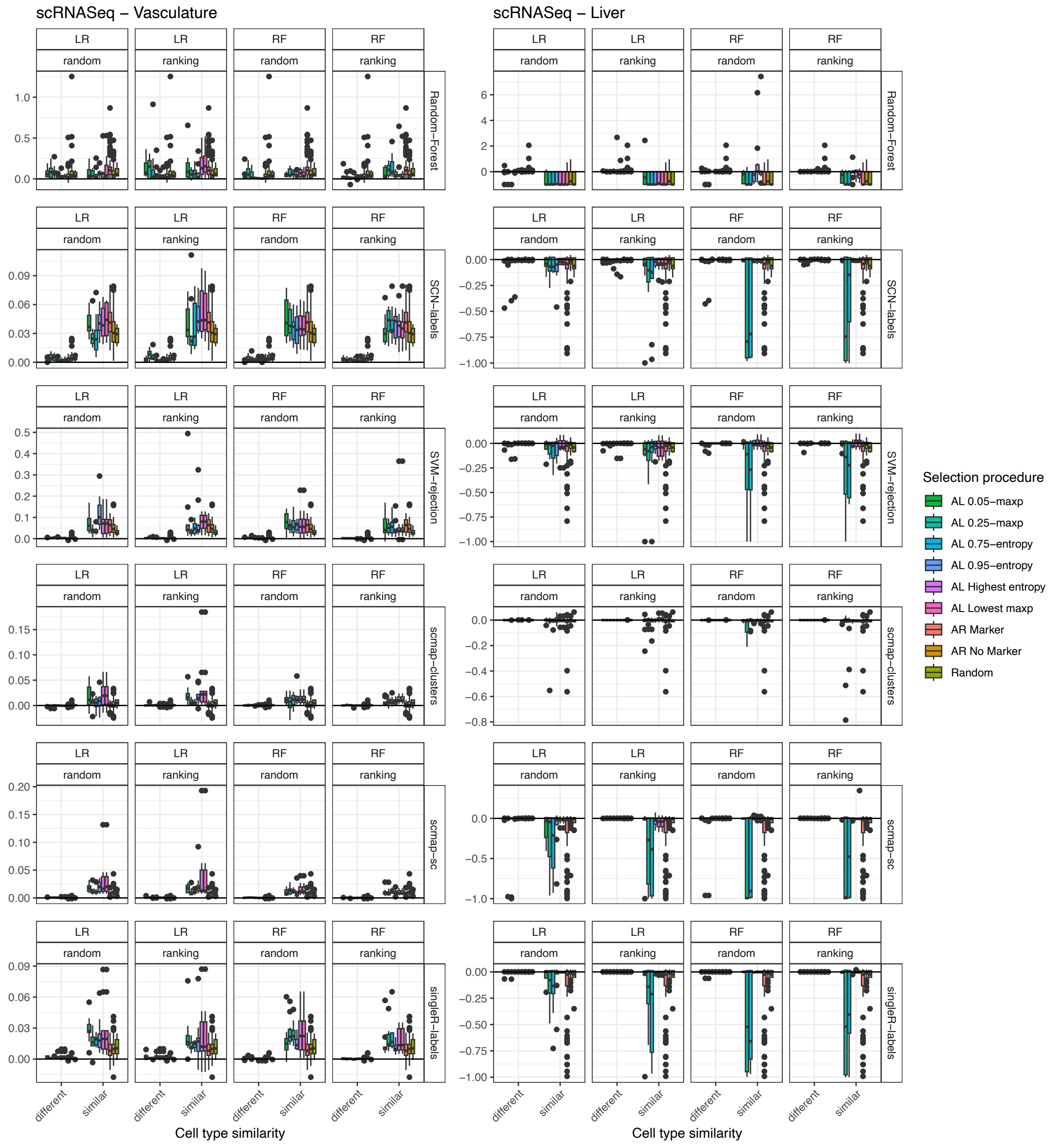

S. Figure 26. Effect of dataset imbalance on sensitivity. Same as S. Figure 25 for the tabula vasculature and liver atlas datasets. Source data are provided on zenodo: https://doi.org/10.5281/zenodo.10403475.

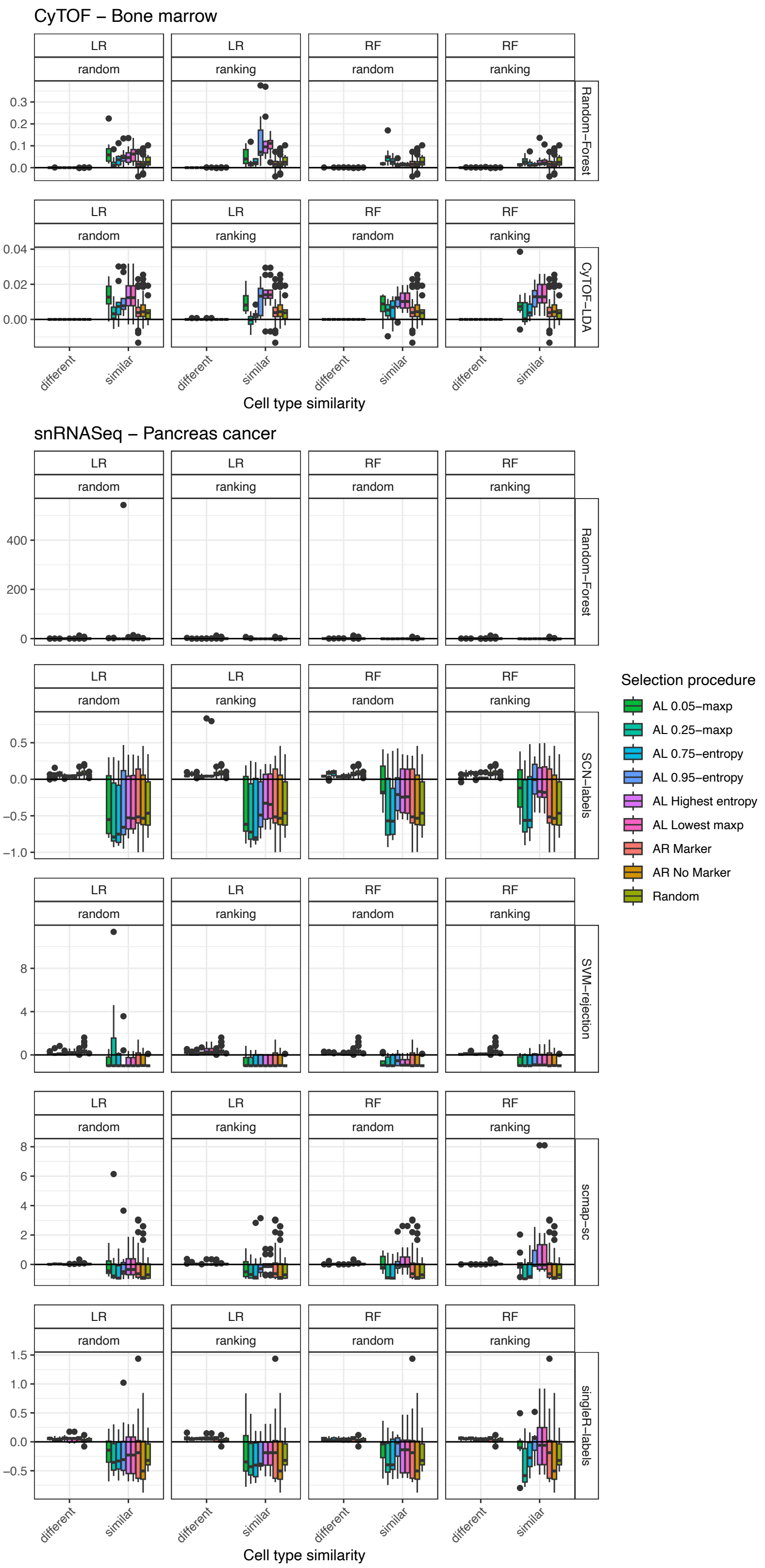
CyTOF – Bone marrow
LR
random
LR
ranking
RF
random
RF
ranking
Random–Forest
CyTOF–LDA
0.3
0.2
0.1
0.0
0.04
0.02
0.00
different
similar
Cell type similarity
snRNASeq – Pancreas cancer
Random–Forest
SCN–labels
SVM–rejection
scmap–sc
singleR–labels
400
200
0
0.5
0.0
−0.5
−1.0
8
4
8
6
4
2
1.5
1.0
Selection procedure
AL 0.05–maxp
AL 0.25–maxp
AL 0.75–entropy
AL 0.95–entropy
AL Highest entropy
AL Lowest maxp
AR Marker
AR No Marker
Random

S. Figure 27. Effect of dataset imbalance on sensitivity. Same as S. Figure 25 for the CyTOF bone marrow and scRNASeq lung cancer cell line dataset. Source data are provided on zenodo: https://doi.org/10.5281/zenodo.10403475.

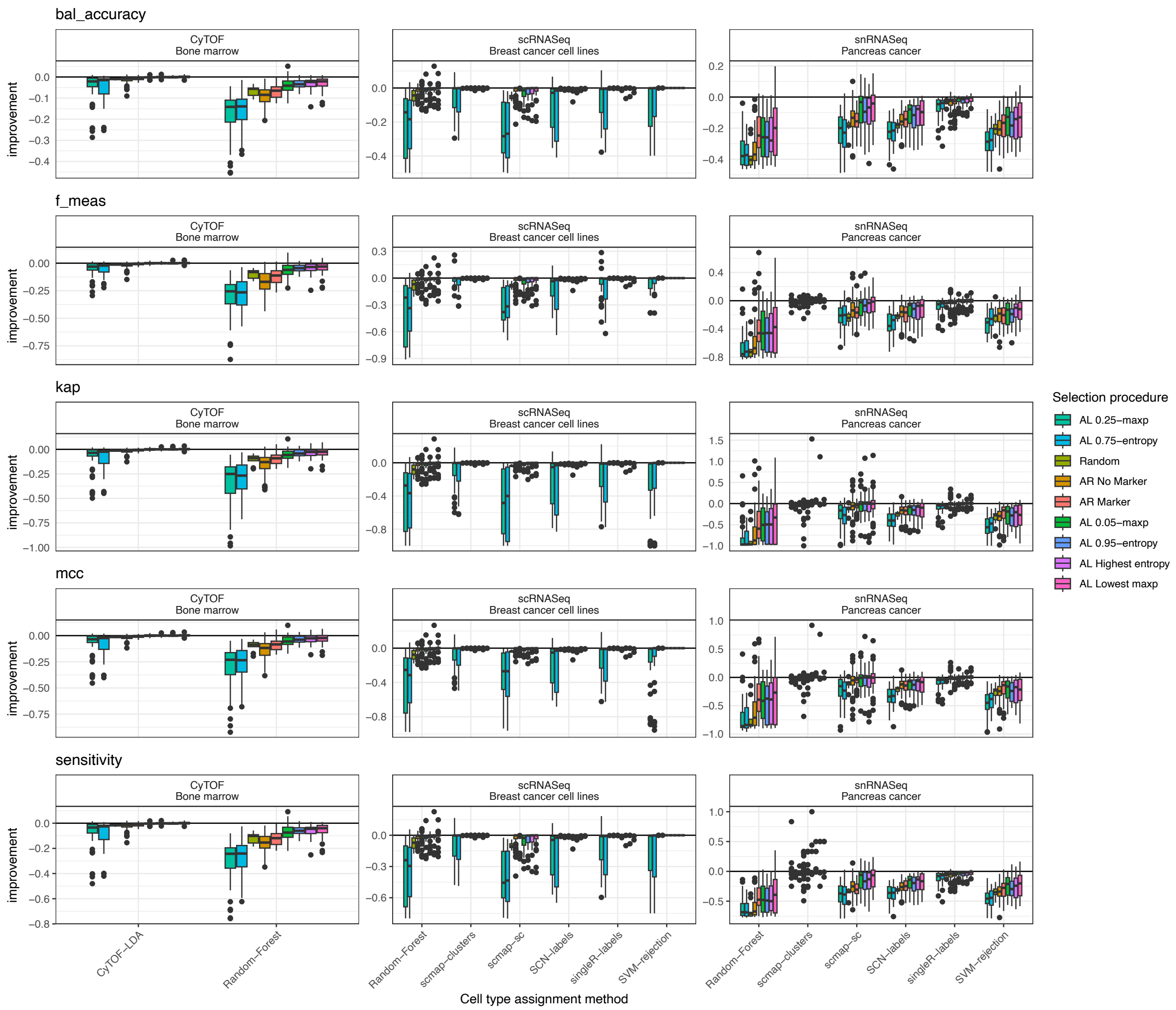

S. Figure 28. Effect of expanded dataset imbalance on classification accuracy for the CyTOF - Bone marrow, scRNASeq - Breast cancer cell line and snRNASeq - Pancreas cancer datasets. Shown is the change for each metric (calculated as accuracy in imbalanced dataset - accuracy in balanced dataset / accuracy in balanced dataset). Each figure is faceted by cohort. Boxplots depict the median as the center line, the boxes define interquartile range (IQR), the whiskers extend up to 1.5 times the IQR and all points depict outliers from this range. Source data are provided on zenodo: https://doi.org/10.5281/zenodo.10403475.

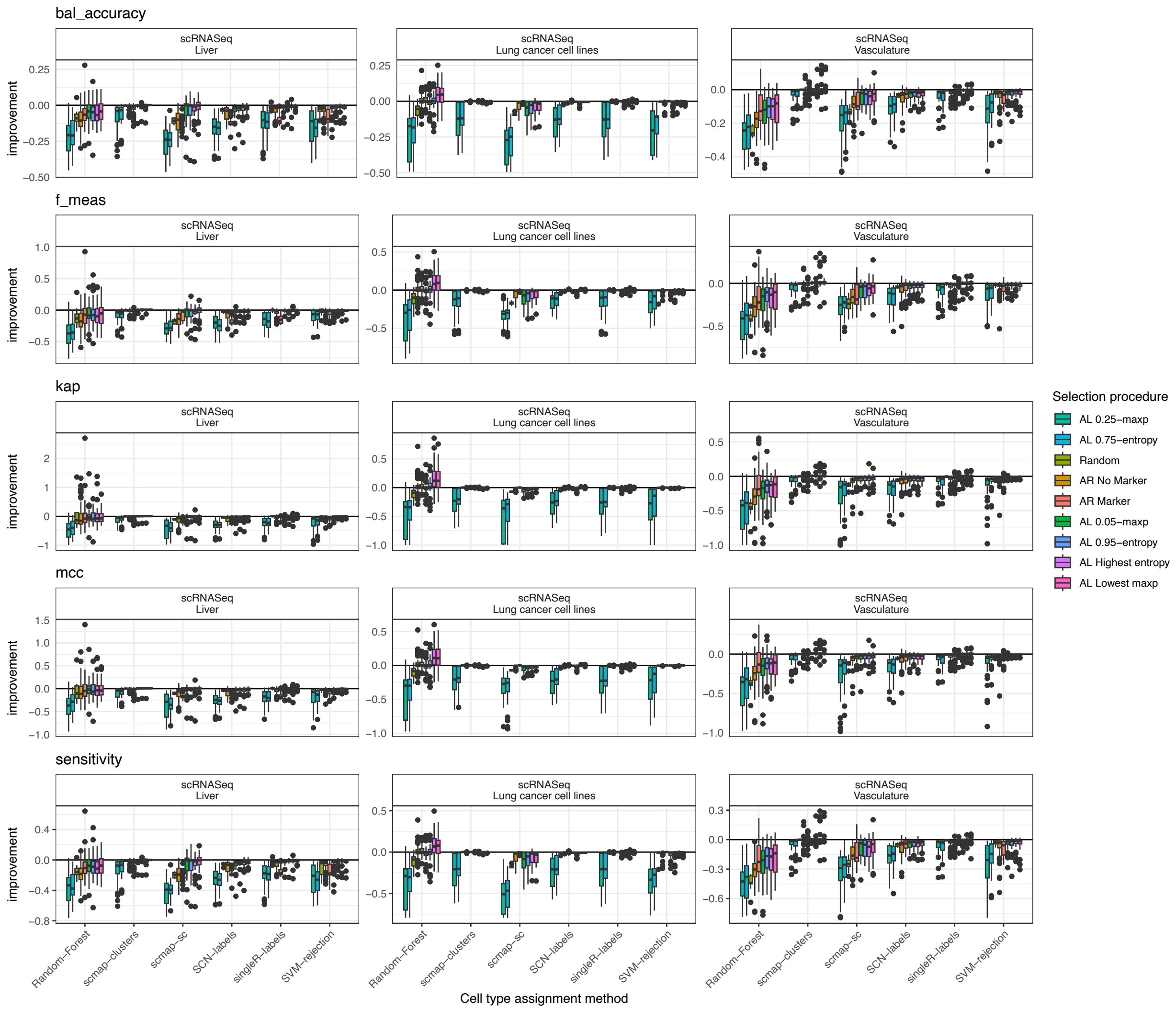

S. Figure 29. Effect of expanded dataset imbalance on classification accuracy for the scRNALung and tabula datasets. Shown is the change for each metric (calculated as accuracy in imbalanced dataset - accuracy in balanced dataset / accuracy in balanced dataset). Source data are provided on zenodo: https://doi.org/10.5281/zenodo.10403475.

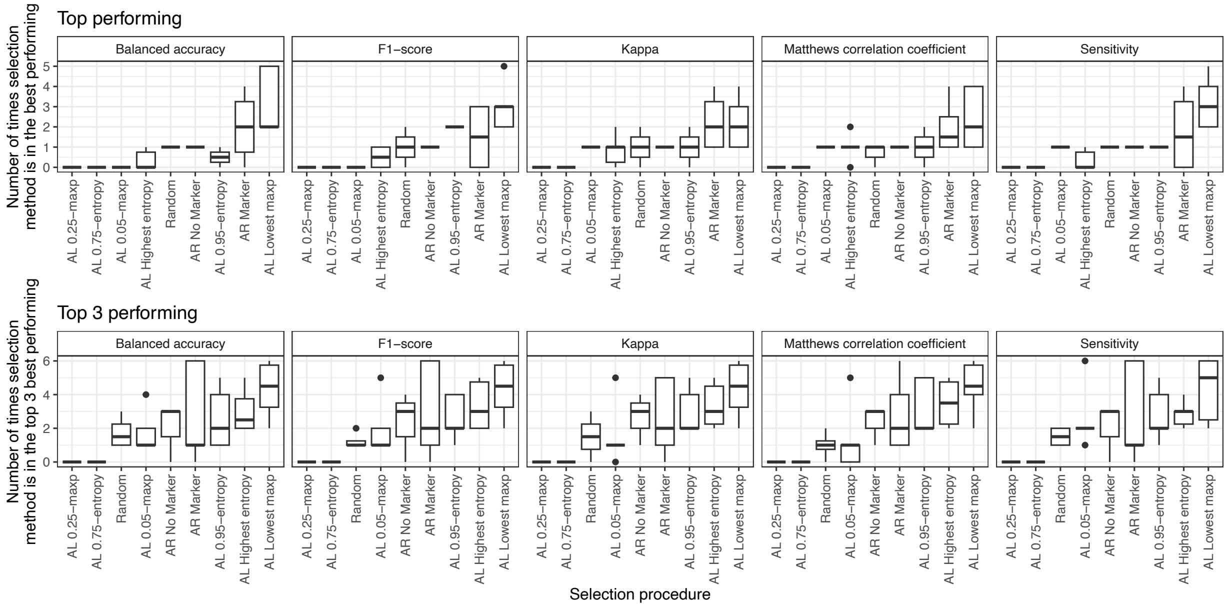

S. Figure 30. Number of times a selection procedure is the top performing method. The average improvement score (as calculated for S. Figures 8 and 9) is calculated for each method, selection procedure, dataset and metric. The number of times each selection procedure is the best performing (top) or among the best 3 performing (bottom) is shown for each metric. The selection procedures are ordered by the average number of times each method is among the top performing group. Boxplots depict the median as the center line, the boxes define interquartile range (IQR), the whiskers extend up to 1.5 times the IQR and all points depict outliers from this range. Source data are provided on zenodo: https://doi.org/10.5281/zenodo.10403475.

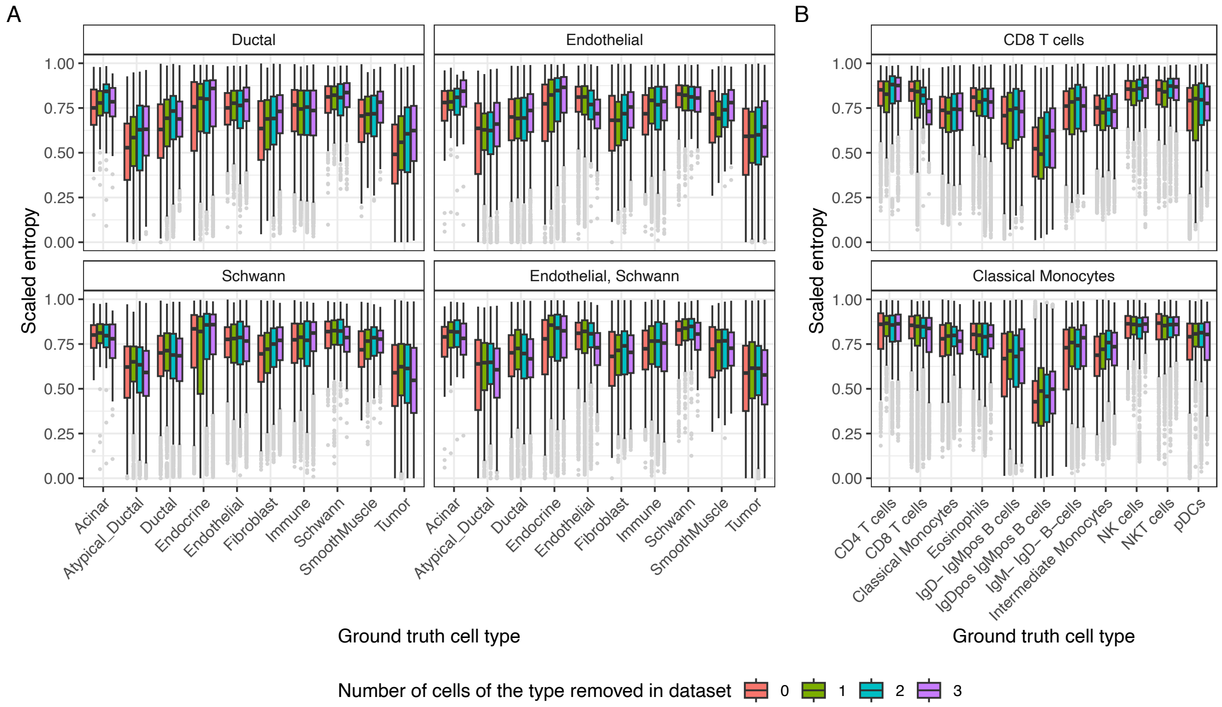

S. Figure 31. Effect of removing cells using a random forest classifier. Shown are entropy values for all cells not in the initial training set of 20 cells. The values were calculated using the predicted probabilities from the active learning classifier after it was trained on the initial dataset. Boxplots are filled by the number of cells present of a particular type (shown in the plot title), while the x axis shows the ground truth cell type label. **A)** scRNASeq dataset with a random forest model. **B)** CyTOF dataset using a random forest model. As entropy is bounded by the total number of classes, the entropy values depicted were scaled by the maximum possible value for each experiment. Shown are the results across the 10 different train test splits. Boxplots depict the median as the center line, the boxes define interquartile range (IQR), the whiskers extend up to 1.5 times the IQR and all points depict outliers from this range. Source data are provided on zenodo: https://doi.org/10.5281/zenodo.10403475.

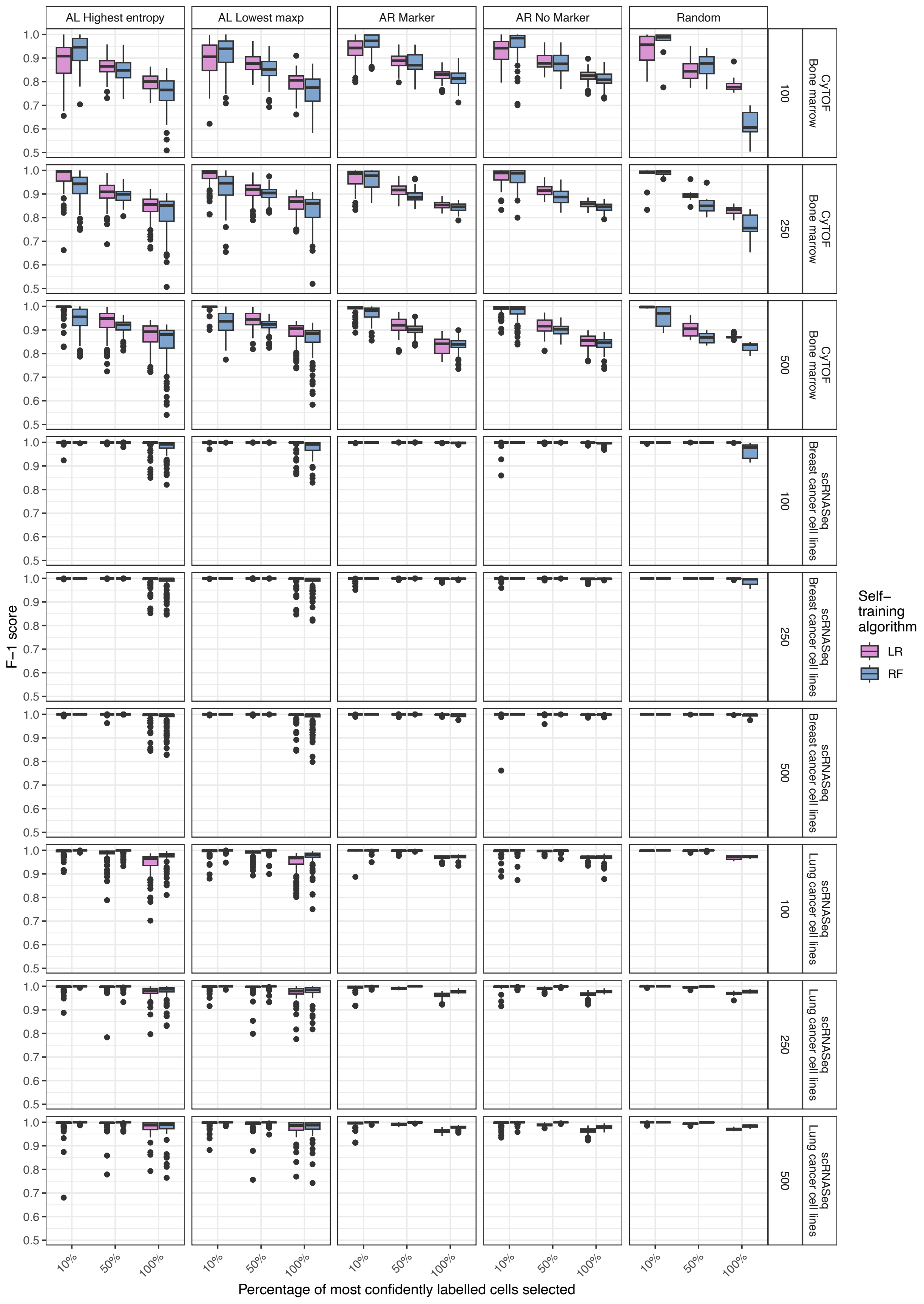
AL Highest entropy
AL Lowest maxp
AR Marker
AR No Marker
Random
CyTOF Bone marrow
scRNASeq Breast cancer cell lines
scRNASeq Lung cancer cell lines
100
250
500
F−1 score
1.0
0.9
0.8
0.7
0.6
0.5
10%
50%
100%
Percentage of most confidently labelled cells selected
Self−training algorithm
LR
RF

S. Figure 32. Accuracy of self-training. Shown is the F1-score for the logistic regression and random forest self-trainers for the CyTOF, breast cancer and lung cancer cell lines. Boxplots depict the median as the center line, the boxes define interquartile range (IQR), the whiskers extend up to 1.5 times the IQR and all points depict outliers from this range. Source data are provided on zenodo: https://doi.org/10.5281/zenodo.10403475.

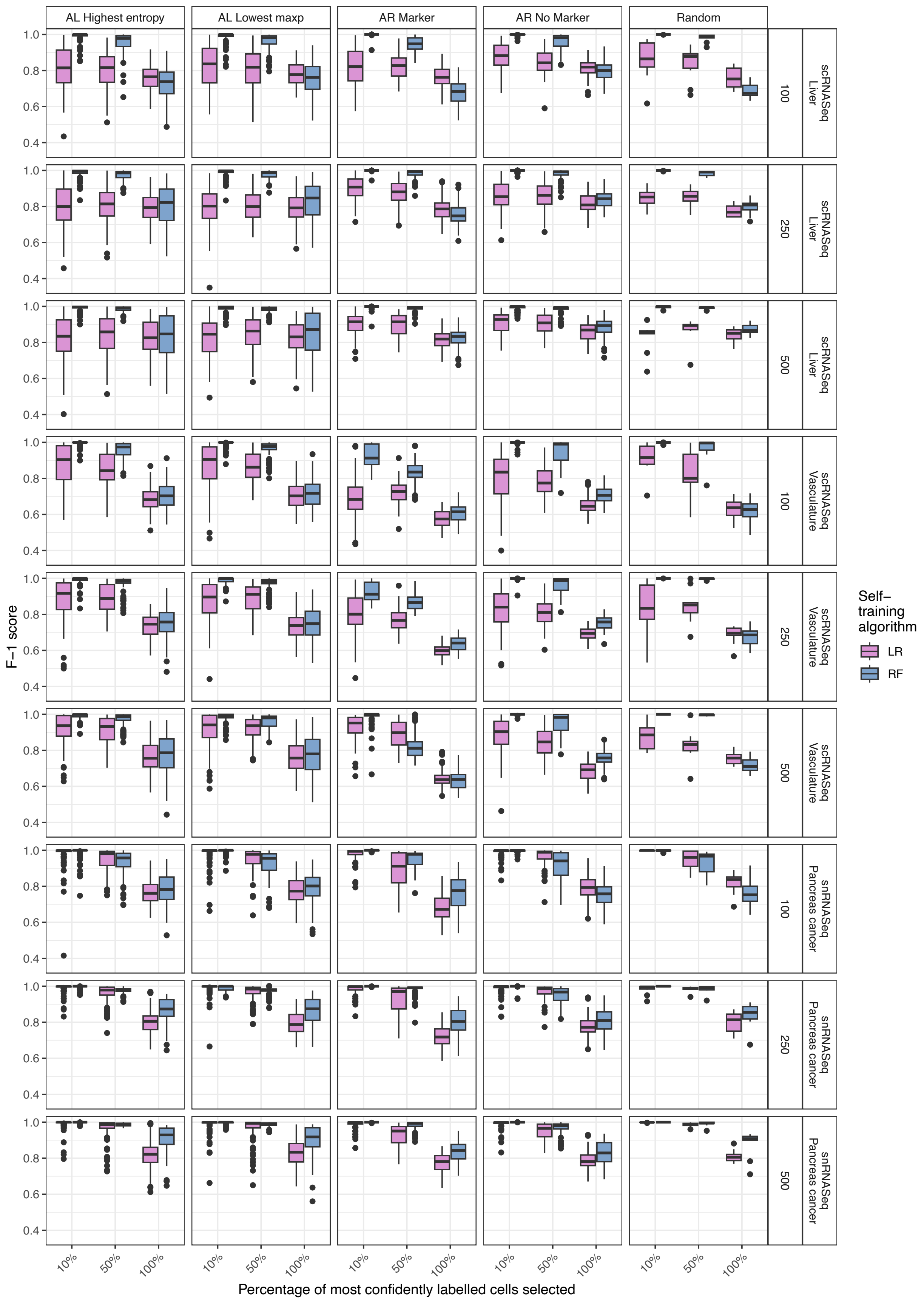

AL Highest entropy
AL Lowest maxp
AR Marker
AR No Marker
Random
scRNASeq Liver
scRNASeq Vasculature
snRNASeq Pancreas cancer
100
250
500
F−1 score
1.0
0.8
0.6
0.4
10%
50%
100%
Percentage of most confidently labelled cells selected
Self−training algorithm
LR
RF

S. Figure 33. Accuracy of self-training. Shown is the F1-score for the logistic regression and random forest self-trainers for the scRNASeq liver and vasculature, and snRNASeq pancreas cancer datasets. Boxplots depict the median as the center line, the boxes define interquartile range (IQR), the whiskers extend up to 1.5 times the IQR and all points depict outliers from this range. Source data are provided on zenodo: https://doi.org/10.5281/zenodo.10403475.

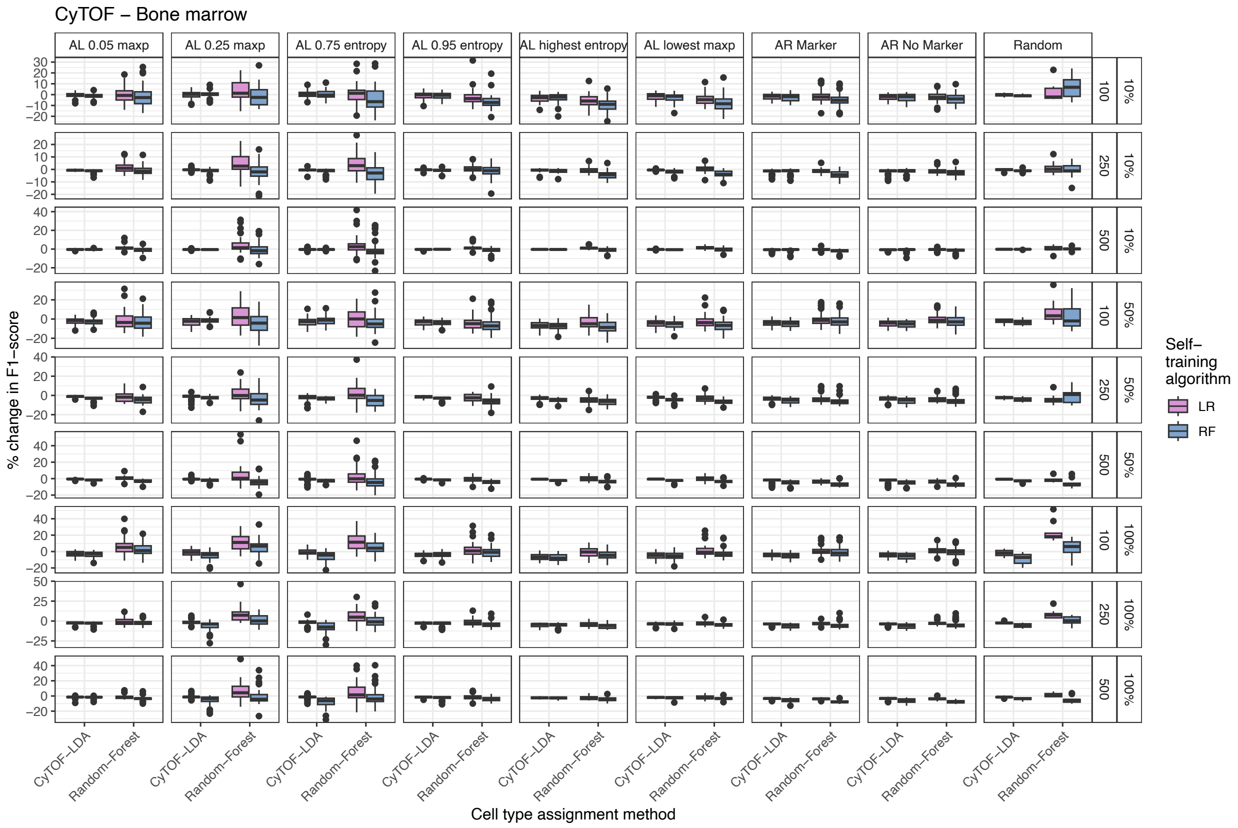

S. Figure 34. Self-training performance boost for the CyTOF Bone marrow cohort. Shown is the improvement in F1-score when self-trained data is included in the dataset. The columns depict the selection method for the initial dataset, while the rows depict the number of cells annotated with ground truth values and the percentage of most confidently labeled cells included. Boxplots depict the median as the center line, the boxes define interquartile range (IQR), the whiskers extend up to 1.5 times the IQR and all points depict outliers from this range. Source data are provided on zenodo: https://doi.org/10.5281/zenodo.10403475.

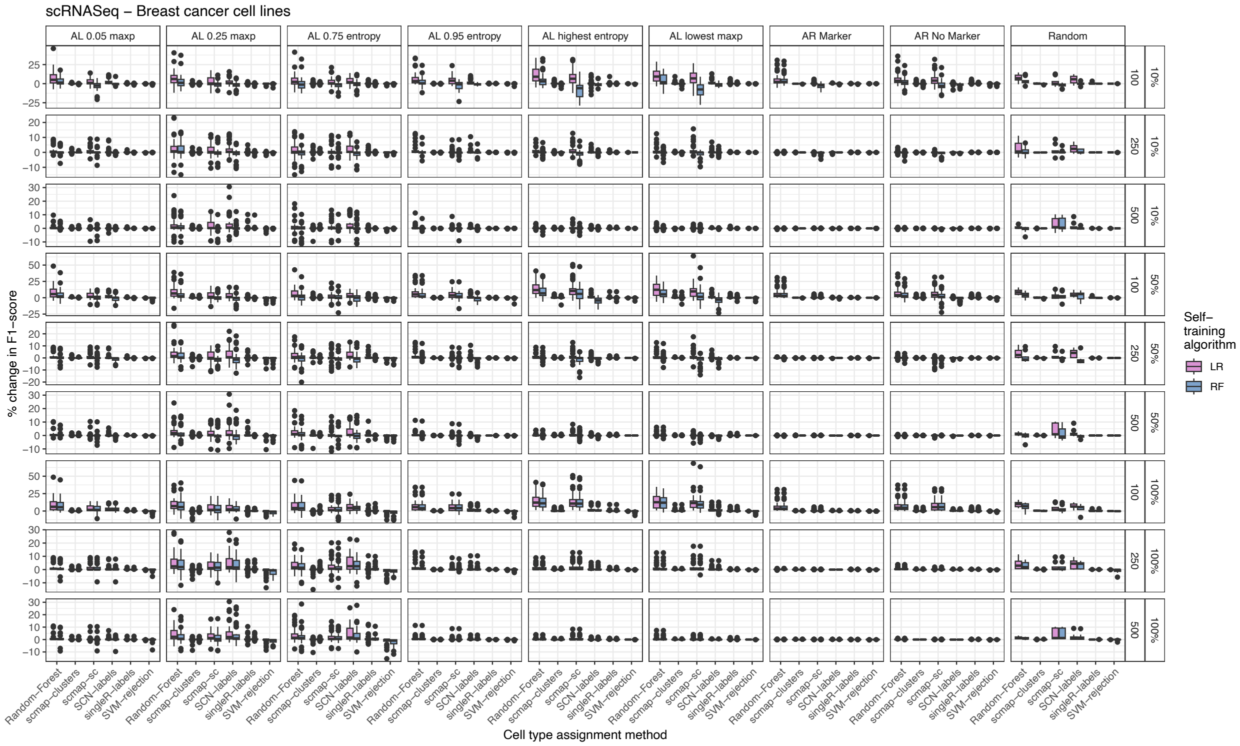

S. Figure 35. Self-training performance boost for the scRNASeq breast cancer cell line cohort. Shown is the improvement in F1-score when self-trained data is included in the dataset. The columns depict the selection method for the initial dataset, while the rows depict the number of cells annotated with ground truth values and the percentage of most confidently labelled cells included. Boxplots depict the median as the center line, the boxes define interquartile range (IQR), the whiskers extend up to 1.5 times the IQR and all points depict outliers from this range. Source data are provided on zenodo: https://doi.org/10.5281/zenodo.10403475.

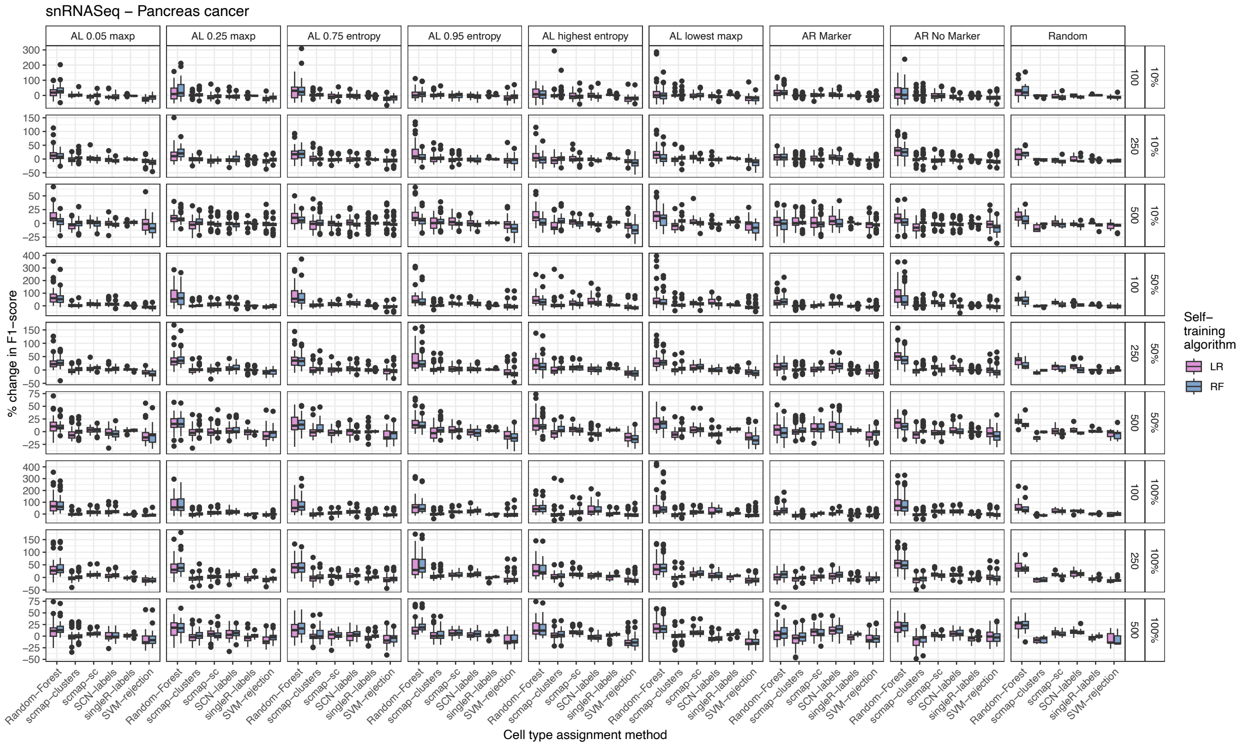

S. Figure 36. Self-training performance boost for the snRNASeq pancreas cancer cohort. Shown is the improvement in F1-score when self-trained data is included in the dataset. The columns depict the selection method for the initial dataset, while the rows depict the number of cells annotated with ground truth values and the percentage of most confidently labelled cells included. Boxplots depict the median as the center line, the boxes define interquartile range (IQR), the whiskers extend up to 1.5 times the IQR and all points depict outliers from this range. Source data are provided on zenodo: https://doi.org/10.5281/zenodo.10403475.

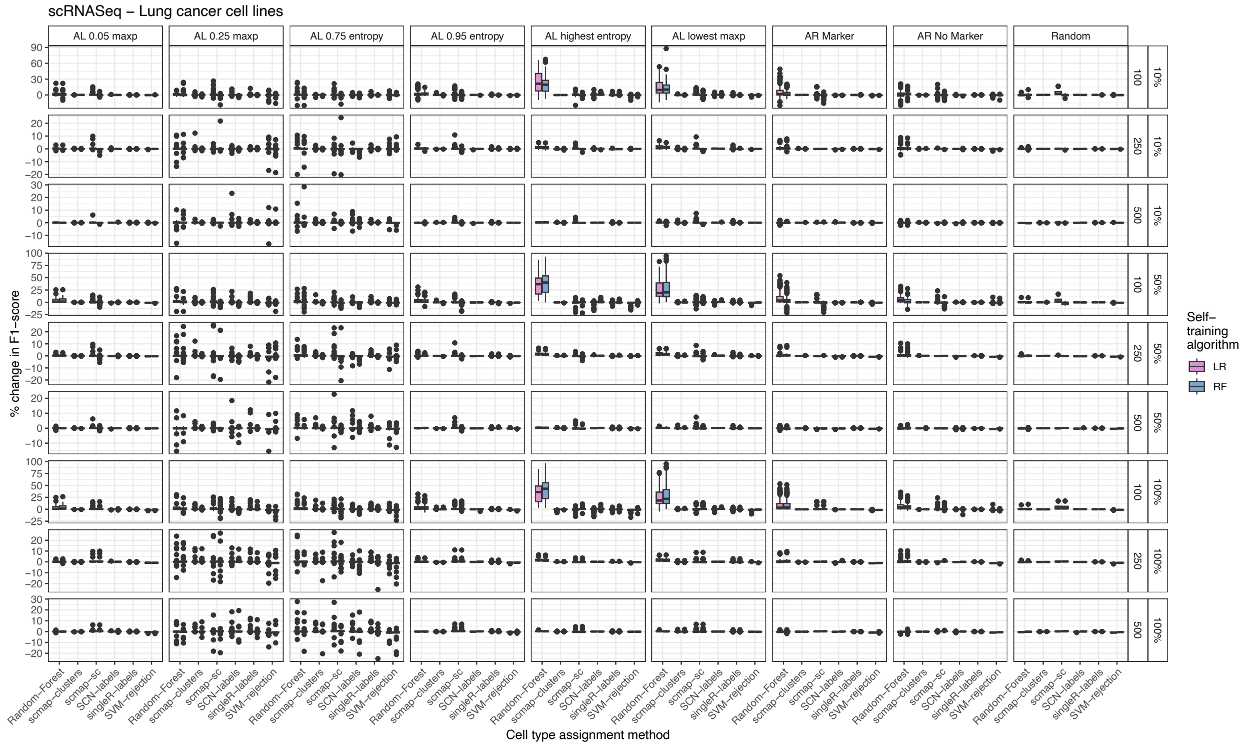

S. Figure 37. Self-training performance boost for the scRNASeq lung cancer cell line cohort. Shown is the improvement in F1-score when self-trained data is included in the dataset. The columns depict the selection method for the initial dataset, while the rows depict the number of cells annotated with ground truth values and the percentage of most confidently labeled cells included. Boxplots depict the median as the center line, the boxes define interquartile range (IQR), the whiskers extend up to 1.5 times the IQR and all points depict outliers from this range. Source data are provided on zenodo: https://doi.org/10.5281/zenodo.10403475.

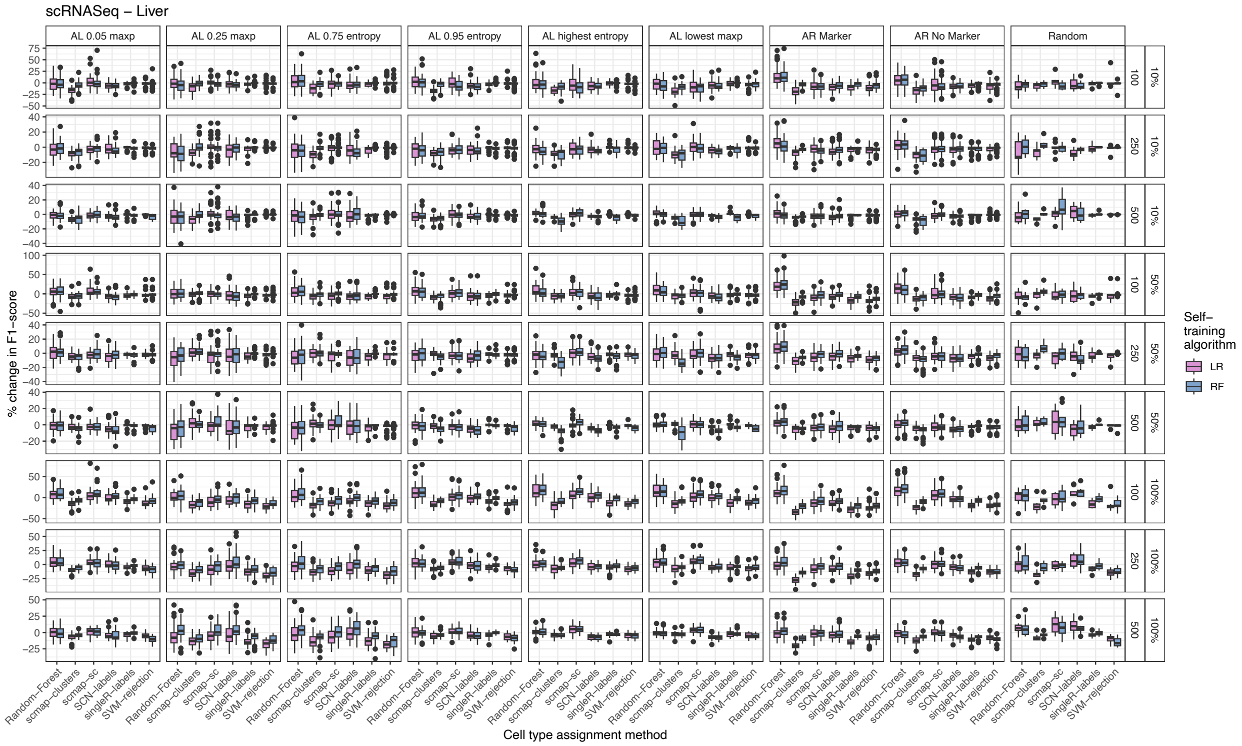

S. Figure 38. Self-training performance boost for the scRNASeq liver cohort. Shown is the improvement in F1-score when self-trained data is included in the dataset. The columns depict the selection method for the initial dataset, while the rows depict the number of cells annotated with ground truth values and the percentage of most confidently labeled cells included. Boxplots depict the median as the center line, the boxes define interquartile range (IQR), the whiskers extend up to 1.5 times the IQR and all points depict outliers from this range. Source data are provided on zenodo: https://doi.org/10.5281/zenodo.10403475.

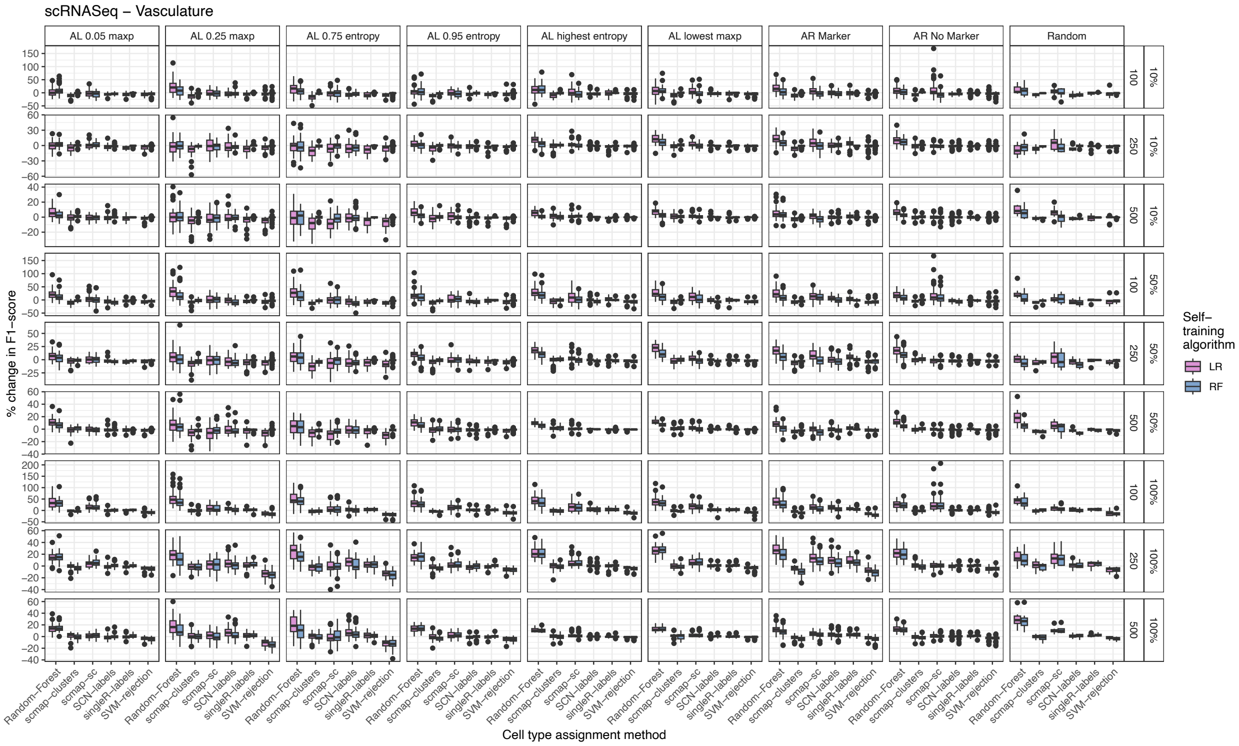

S. Figure 39. Self-training performance boost for the scRNASeq vasculature cohort. Shown is the improvement in F1-score when self-trained data is included in the dataset. The columns depict the selection method for the initial dataset, while the rows depict the number of cells annotated with ground truth values and the percentage of most confidently labeled cells included. Boxplots depict the median as the center line, the boxes define interquartile range (IQR), the whiskers extend up to 1.5 times the IQR and all points depict outliers from this range. Source data are provided on zenodo: https://doi.org/10.5281/zenodo.10403475.

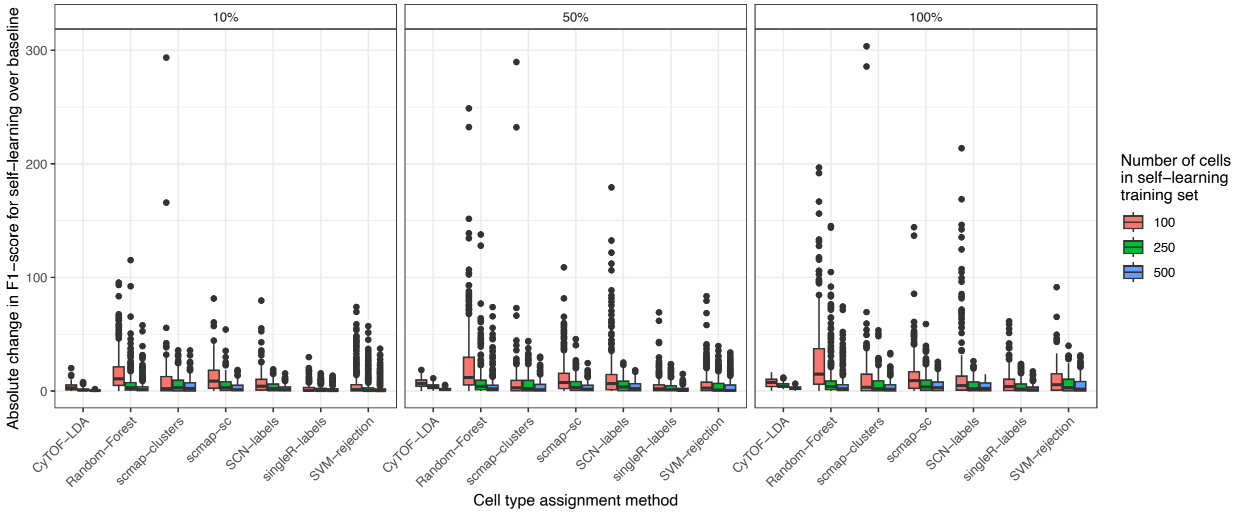

S. Figure 40. The effect of self-training is limited to scenarios with a low number of training cells. Shown is the absolute change in F1-score between the classifier performance

when self-trained cell type labels are included and excluded for each cell type assignment method using a different number of cells in the initial self-training dataset. The panels are faceted by the number of self-trained cells included, e.g. 10% equates to including the 10% most confidently labeled cells in the training dataset. Boxplots depict the median as the center line, the boxes define interquartile range (IQR), the whiskers extend up to 1.5 times the IQR and all points depict outliers from this range. Source data are provided on zenodo: https://doi.org/10.5281/zenodo.10403475.
