# Supplementary material for: The impacts of active and self-supervised learning on efficient annotation of single-cell expression data
Source: Nat Commun. 2024 Feb 3;15:1014. doi: 10.1038/s41467-024-45198-y (PMC10837127; doi:10.1038/s41467-024-45198-y)
Supplement: Supplementary file 3 — Reporting Summary [file 41467_2024_45198_MOESM3_ESM.pdf]

nature portfolio

Corresponding author(s): Kieran Campbell

Last updated by author(s): Dec 19, 2023

Reporting Summary

Nature Portfolio wishes to improve the reproducibility of the work that we publish. This form provides structure for consistency and transparency in reporting. For further information on Nature Portfolio policies, see our Editorial Policies and the Editorial Policy Checklist.

Statistics

For all statistical analyses, confirm that the following items are present in the figure legend, table legend, main text, or Methods section.

n/a	Confirmed	
☐	☒	The exact sample size (*n*) for each experimental group/condition, given as a discrete number and unit of measurement
☐	☒	A statement on whether measurements were taken from distinct samples or whether the same sample was measured repeatedly
☒	☐	The statistical test(s) used AND whether they are one- or two-sided<br>*Only common tests should be described solely by name; describe more complex techniques in the Methods section.*
☒	☐	A description of all covariates tested
☒	☐	A description of any assumptions or corrections, such as tests of normality and adjustment for multiple comparisons
☐	☒	A full description of the statistical parameters including central tendency (e.g. means) or other basic estimates (e.g. regression coefficient) AND variation (e.g. standard deviation) or associated estimates of uncertainty (e.g. confidence intervals)
☒	☐	For null hypothesis testing, the test statistic (e.g. *F*, *t*, *r*) with confidence intervals, effect sizes, degrees of freedom and *P* value noted<br>*Give P values as exact values whenever suitable.*
☒	☐	For Bayesian analysis, information on the choice of priors and Markov chain Monte Carlo settings
☒	☐	For hierarchical and complex designs, identification of the appropriate level for tests and full reporting of outcomes
☐	☒	Estimates of effect sizes (e.g. Cohen's *d*, Pearson's *r*), indicating how they were calculated

Our web collection on statistics for biologists contains articles on many of the points above.

Software and code

Policy information about availability of computer code

Data collection	The CyTOF bone marrow dataset was downloaded using the HDCytoData R package (version 1.22). All other datasets were downloaded manually.
Data analysis	All software packages used are listed in the Pipfile and the Docker file used to create the container, and are available on our github repository and archived on zenodo: https://github.com/camlab-bioml/active-learning-benchmarking and https://zenodo.org/doi/10.5281/zenodo.10397828

For manuscripts utilizing custom algorithms or software that are central to the research but not yet described in published literature, software must be made available to editors and reviewers. We strongly encourage code deposition in a community repository (e.g. GitHub). See the Nature Portfolio guidelines for submitting code & software for further information.

Data

Policy information about availability of data

All manuscripts must include a data availability statement. This statement should provide the following information, where applicable:

- Accession codes, unique identifiers, or web links for publicly available datasets
- A description of any restrictions on data availability
- For clinical datasets or third party data, please ensure that the statement adheres to our policy

Breast cancer dataset was downloaded from: https://figshare.com/articles/dataset/Single_Cell_Breast_Cancer_cell-line_Atlas/15022698

The lung cancer cell line dataset was downloaded from: https://github.com/LuyiTian/sc_mixology
The tabula sapiens vasculature dataset was downloaded from: https://figshare.com/articles/dataset/Tabula_Sapiens_release_1_0/14267219
The liver cell atlas dataset was downloaded from: https://www.livercellatlas.org
The pancreatic cancer snRNASeq dataset was downloaded from: https://singlecell.broadinstitute.org/single_cell/study/SCP1089/human-treatment-naive-pdac-snuc-seq
The CyTOF bone marrow dataset was downloaded using the HDCytoData R package.
Cell type markers were either taken from the supplementary data of the original publication or downloaded from pangaloDB at https://panglaodb.se.

Research involving human participants, their data, or biological material

Policy information about studies with human participants or human data. See also policy information about sex, gender (identity/presentation), and sexual orientation and race, ethnicity and racism.

Reporting on sex and gender	n/a
Reporting on race, ethnicity, or other socially relevant groupings	n/a
Population characteristics	n/a
Recruitment	n/a
Ethics oversight	n/a

Note that full information on the approval of the study protocol must also be provided in the manuscript.

Field-specific reporting

Please select the one below that is the best fit for your research. If you are not sure, read the appropriate sections before making your selection.

[x] Life sciences [ ] Behavioural & social sciences [ ] Ecological, evolutionary & environmental sciences

For a reference copy of the document with all sections, see nature.com/documents/nr-reporting-summary-flat.pdf

Life sciences study design

All studies must disclose on these points even when the disclosure is negative.

Sample size	A total of 6 single cell datasets were analyzed in total for this study. This is designed to balance coverage of cellular phenotypes across tissues and cell lineages while maintaining computational feasibility given the available resources.
Data exclusions	Beyond dataset downsampling no data was excluded.
Replication	All experiments were replicated across the 6 datasets as well as 10 times within each dataset across different train-test splits.
Randomization	Cells were randomly split into a train or test set. Beyond this the analysis did not require further randomization of the data.
Blinding	Blinding is not relevant for this work because it is not a case control study but rather an investigation of algorithmic properties of cell type assignment.

Reporting for specific materials, systems and methods

We require information from authors about some types of materials, experimental systems and methods used in many studies. Here, indicate whether each material, system or method listed is relevant to your study. If you are not sure if a list item applies to your research, read the appropriate section before selecting a response.

Materials & experimental systems

n/a	Involved in the study	
x		Antibodies
x		Eukaryotic cell lines
x		Palaeontology and archaeology
x		Animals and other organisms
x		Clinical data
x		Dual use research of concern
x		Plants

Methods

n/a	Involved in the study	
x		ChIP-seq
x		Flow cytometry
x		MRI-based neuroimaging

Plants

Seed stocks	*Report on the source of all seed stocks or other plant material used. If applicable, state the seed stock centre and catalogue number. If plant specimens were collected from the field, describe the collection location, date and sampling procedures.*
Novel plant genotypes	*Describe the methods by which all novel plant genotypes were produced. This includes those generated by transgenic approaches, gene editing, chemical/radiation-based mutagenesis and hybridization. For transgenic lines, describe the transformation method, the number of independent lines analyzed and the generation upon which experiments were performed. For gene-edited lines, describe the editor used, the endogenous sequence targeted for editing, the targeting guide RNA sequence (if applicable) and how the editor was applied.*
Authentication	*Describe any authentication procedures for each seed stock used or novel genotype generated. Describe any experiments used to assess the effect of a mutation and, where applicable, how potential secondary effects (e.g. second site T-DNA insertions, mosiacism, off-target gene editing) were examined.*
